# Exploring bet-hedging in *Salmonella enterica* serovar Typhimurium with a dual reporter strain

**DOI:** 10.1128/msystems.00177-26

**Published:** 2026-05-18

**Authors:** Michelle I. Gerber, Murugesan Sivaranjani, Kyle Vincent, Samantha Jelfimow, Dakoda J. Herman, Melissa B. Palmer, Chinenye R. Nnajide, Haley Sanderson, Jessica Sparrow, Brandon M. Waddell, Neeraj Dhar, Cheng-Wei Wu, Aaron P. White

**Affiliations:** 1 Vaccine and Infectious Disease Organization (VIDO), University of Saskatchewan, Saskatoon, Saskatchewan, Canada; 2 Department of Biochemistry, Microbiology and Immunology, University of Saskatchewan 7235, Saskatoon, Saskatchewan, Canada; 3 Department of Veterinary Biomedical Sciences, Western College of Veterinary Medicine, University of Saskatchewan, Saskatoon, Saskatchewan, Canada; University of Minnesota Twin Cities, Minneapolis, Minnesota, USA

**Keywords:** *Salmonella*, fluorescent reporter, bet-hedging, type three secretion, CsgD, biofilm, planktonic cells, bistable gene expression, *C. elegans*

## Abstract

Non-typhoidal *Salmonella* are a leading cause of foodborne illness. These pathogens cycle between infecting hosts and persisting in the environment as biofilms. Unfortunately, this transition stage is not well understood. In non-typhoidal *Salmonella,* bistable synthesis of the master biofilm regulator CsgD results in a population split into CsgD-positive biofilm aggregates that synthesize a protective extracellular matrix and CsgD-negative single cells that synthesize the *Salmonella* pathogenicity island 1 type III secretion system (SPI-1 T3SS), which facilitates host cell invasion. We hypothesize that this is a bet-hedging strategy evolved to improve transmission, providing a way for NTS to cause disease immediately (single cells) or persist over extended periods (biofilms). We built a fluorescent reporter strain of *S*. Typhimurium to simultaneously track biofilm^+^ cells (GFP) and SPI-1 T3SS^+^ cells (mCherry) to better understand the dynamics of bet-hedging. Four *S*. Typhimurium cell populations were quantified in an *in vitro* flask model of biofilm development: CsgD^+^/SPI-1⁻, CsgD⁻/SPI-1^+^, CsgD^+^/SPI-1^+^, and CsgD⁻/SPI-1⁻. We demonstrate that *Salmonella* population splitting can occur *in vivo* during infection of *Caenorhabditis elegans*. At early stages of infection, the worm intestine was mainly dominated by SPI-1 T3SS^+^ *Salmonella* cells, while at later time points, both SPI-1 T3SS^+^ cells and biofilm^+^ cells are present. The use of the *S*. Typhimurium dual reporter will allow us to track and better understand the importance of bet-hedging in the *Salmonella* lifecycle.

**IMPORTANCE:**

*Salmonella* strains that cause gastroenteritis remain a leading cause of illness and deaths related to foodborne disease. These bacteria follow a cyclical life cycle (i.e., host → environment → host) that is key to their continued success as pathogens. Here, we built a dual reporter strain to study the infectious single cells and biofilm cell types of *Salmonella enterica* serovar Typhimurium, one of the most common gastroenteritis-causing serovars worldwide. Studying infectious and persistent cell types simultaneously and how they interact with host tissues *in vivo* will provide new insights into the *Salmonella* lifecycle and will be important to develop interventions that reduce outbreaks and disease.

## INTRODUCTION

It has long been established that genetically identical bacterial cells exposed to the same conditions can respond heterogeneously. Unlike laboratory settings, bacteria living in natural environments experience highly fluctuating conditions, making it difficult for cells to respond quickly. To mitigate the risk of unpredictability, bacteria can generate two or more phenotypically distinct subpopulations that are differentially adapted to survive, a phenomenon called bet-hedging (1–6). Many enteric or diarrhea-causing pathogens have a host–environment–host cyclical lifestyle, which imparts a survival risk to cells entering the natural environment because of the unknown physical conditions and host availability (7–10). We and others have hypothesized that *Salmonella* employs a bet-hedging strategy in its lifecycle to improve the chance of survival and transmission.

*Salmonella* strains that cause gastroenteritis remain one of the top enteric pathogens that cause foodborne illness and death (11). These bacteria, collectively called non-typhoidal *Salmonella* (NTS), have a broad host range and cause a self-limiting diarrheal disease in humans, mammals, and birds. Susceptible hosts become infected with NTS by consuming contaminated food or water, or by the fecal–oral route. In the intestine, *Salmonella* uses type III secretion systems (T3SS) to invade host intestinal epithelial cells and survive and replicate inside macrophages (12, 13). The primary pathology of an NTS infection is inflammation and diarrhea, which facilitates bacterial shedding and dissemination into the environment, where biofilms are thought to play a role in survival (8, 10, 14–16). In *Salmonella,* biofilm formation is controlled by the transcription factor CsgD, which regulates production of major biofilm components such as proteinaceous curli fimbriae and the polysaccharide polymer, cellulose (17). Previous research has shown that synthesis of CsgD is bistable (18, 19). *Salmonella enterica* serovar Typhimurium grown in an *in vitro* flask model of biofilm development split the population of cells into aggregates and planktonic cells, and the two cell types were found to differentially express more than 1,800 genes (19). The aggregated cells synthesize high levels of CsgD over time, while the planktonic cells do not (19). In contrast, the planktonic cells expressed many virulence genes and were found to synthesize a functional *Salmonella* pathogenicity island I (SPI-1) T3SS (19). To test whether these cells were truly infectious, mice were infected with equal numbers of aggregates and planktonic cells in a competitive infection assay, which showed that the planktonic cells had a significant virulence advantage (19).

*Salmonella* population splitting into aggregates and infectious single cells may represent a bet-hedging strategy that is important for the NTS life cycle. In contrast, this strategy is lost or reduced in strains that transmit more directly from person to person (20). We hypothesize that *S*. Typhimurium differentiates into biofilm^+^ and SPI-1 T3SS^+^ cell subpopulations *in vivo*. The simultaneous formation of both cell types in the intestine before release into the environment would prepare *Salmonella* for either immediate (i.e., SPI-1 T3SS^+^ cells) or delayed (biofilm^+^ cells) infection opportunities (19). Although the host intestinal environment does not represent typical biofilm-inducing conditions, we recently found that *S*. Typhimurium 14028 produces curli fimbriae in the cecum and colon of infected mice (21). While CsgD is not thought to be important for overall virulence (22), curli expression has been shown to trigger autoimmunity and arthritis in the infected host (23–25).

In this study, we built a dual fluorescent construct that allows us to track *Salmonella* biofilm and SPI-1 T3SS gene expression simultaneously. We show for the first time that CsgD-mediated population splitting is not a random process and could be a conserved survival strategy for *Salmonella* transmission.

## RESULTS

### Tracking biofilm cells and virulent single cells of *S*. Typhimurium

**Fig 1:**
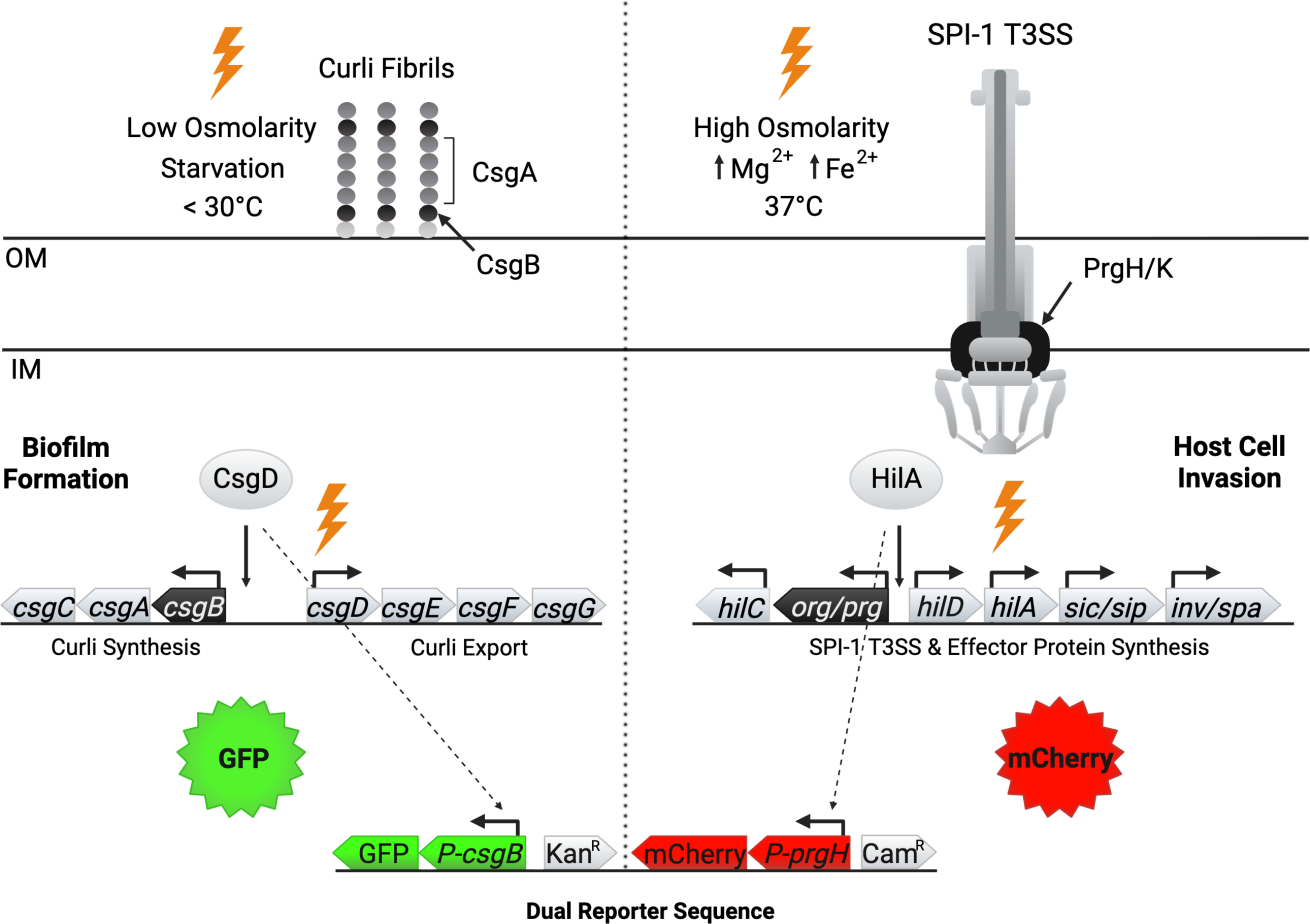
Design of a dual reporter construct to monitor *Salmonella* Typhimurium 14028 population splitting. Schematic of two genetic programs relating to *Salmonella* bet hedging: (1) Biofilm production and assembly of curli fimbriae or (2) virulence—the SPI-1 type III secretion system. The master biofilm regulator protein CsgD is activated by external signals and binds directly to the promoter of *csgB* at the native locus to activate synthesis of an essential biofilm protein polymer called curli. The SPI-1 T3SS transcriptional regulator HilA responds to external cues and binds to the promoter of *prgH* at the native locus to activate synthesis of the SPI-1 T3SS apparatus and various secreted effector proteins. The dual reporter construct is shown at the bottom as it was integrated into the *S*. Typhimurium chromosome; CsgD will bind to the *csgB* promoter to drive transcription of green fluorescent protein (GFP), while HilA will bind to the *prgH* promoter to drive transcription of mCherry. This figure was created using Biorender.

We previously investigated the growth of *S*. Typhimurium 14028 in an *in vitro* flask model for biofilm development (19). The coexistence of biofilm aggregates adapted for persistence and single cells adapted for virulence was intriguing, since the production of biofilm polymers and the SPI-1 type III secretion system are both high-energy-utilizing processes. The production of curli fimbriae is key for biofilm formation, with expression controlled by binding of the transcriptional regulator, CsgD (26). In a previous study, we had constructed a *csgB::GFP* reporter strain to track biofilm formation (19). We also wanted to track SPI-1 T3SS gene expression, but there are multiple proteins that regulate transcription at several key promoters (27). We monitored the expression of four major SPI-1 promoters (*hilD*, *hilA*, *prgH,* and *invF*) during the growth of *S*. Typhimurium in biofilm-inducing media, supplemented with an extract from a continuous culture chemostat (28, 29). All four SPI-1 promoters were activated at similar times during growth, and the *prgH* promoter had the strongest expression (Fig. S1). Therefore, we built a single reporter strain of *S*. Typhimurium with the *prgH* promoter controlling expression of mCherry. PrgH is one of three basal body proteins required for SPI-1 T3SS assembly and requires binding of HilA for activation (30). The two single reporter strains contained a major transcriptional target of either the master biofilm regulator (CsgD) or a master SPI-1 regulator (HilA) (Fig. 1).

### Building dual reporter strains to track cell types using fluorescence

**Fig 2:**
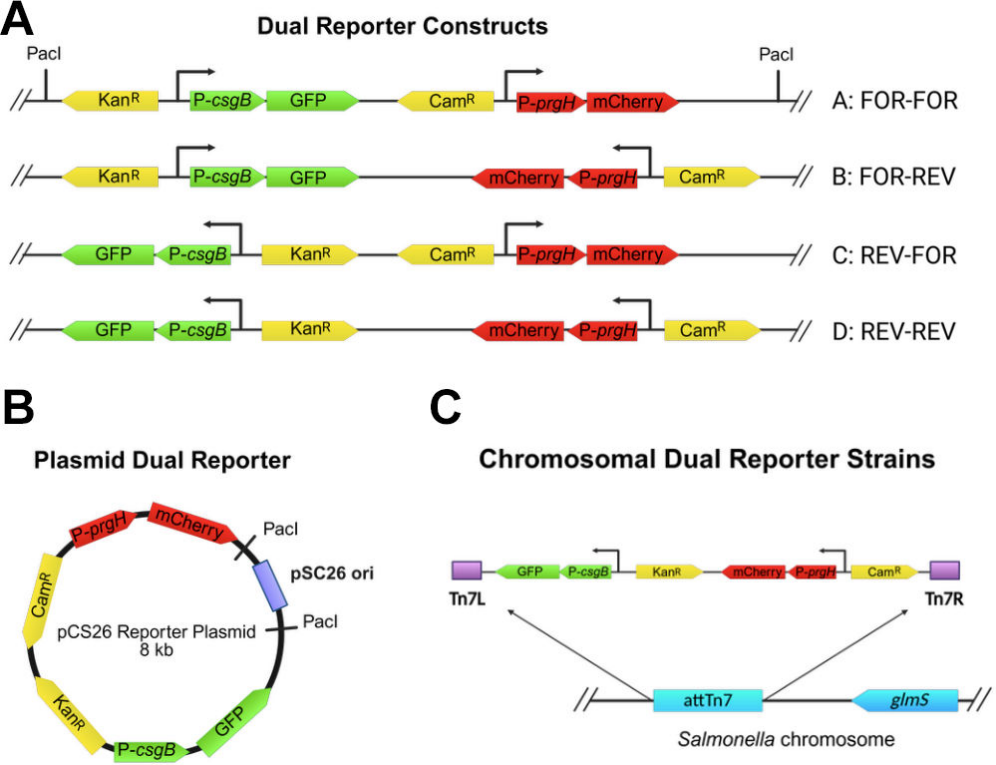
Design and construction of *Salmonella* serovar Typhimurium dual reporter strains. (**A**) Four “dual reporter constructs” were designed with *csgB* and *prgH* promoters facing in different directions: “A” = FOR-FOR; “B” = FOR-REV; “C” = REV-FOR; and “D” = REV-REV. Each reporter construct contains the *aph*(3′) and *cat* genes for selection with kanamycin (Kan) and chloramphenicol (Cam), and *Pac*I sites at the 5′ and 3′ ends for downstream cloning steps. Each dual reporter construct was built using Golden Gate Assembly and cloned between the Tn7L and Tn7R ends in the pUC18R6K-miniTn7T-PacI vector (31). (**B**) Construct “C” was also cloned into pCS26 (32) and used as a “plasmid dual reporter.” (**C**) The pUC18R6K-miniTn7T-PacI vectors were used with pHSG415-*tnsABCD*, which encodes the Tn7 transposition machinery, to insert each construct directionally into the attTn7 site in the *S*. Typhimurium 14028 chromosome, downstream of *glmS,* to generate the “chromosomal dual reporter strains.” The version shown is the “D” construct. This figure was created using Biorender.

We hypothesized that we could join the *csgB::GFP* and *prgH::mCherry* reporter fragments together and track biofilm and virulence gene expression at the same time. Four dual reporter constructs were designed with *csgB* and *prgH* promoters facing different directions (Fig. 2). Each construct was integrated into the attTn7 site in the chromosome of *S*. Typhimurium 14028, downstream of the *glmS* gene, using our established protocol (31). We also built a plasmid-based dual reporter strain of *S*. Typhimurium 14028 as a contingency plan (Fig. 2).

**Fig 3:**
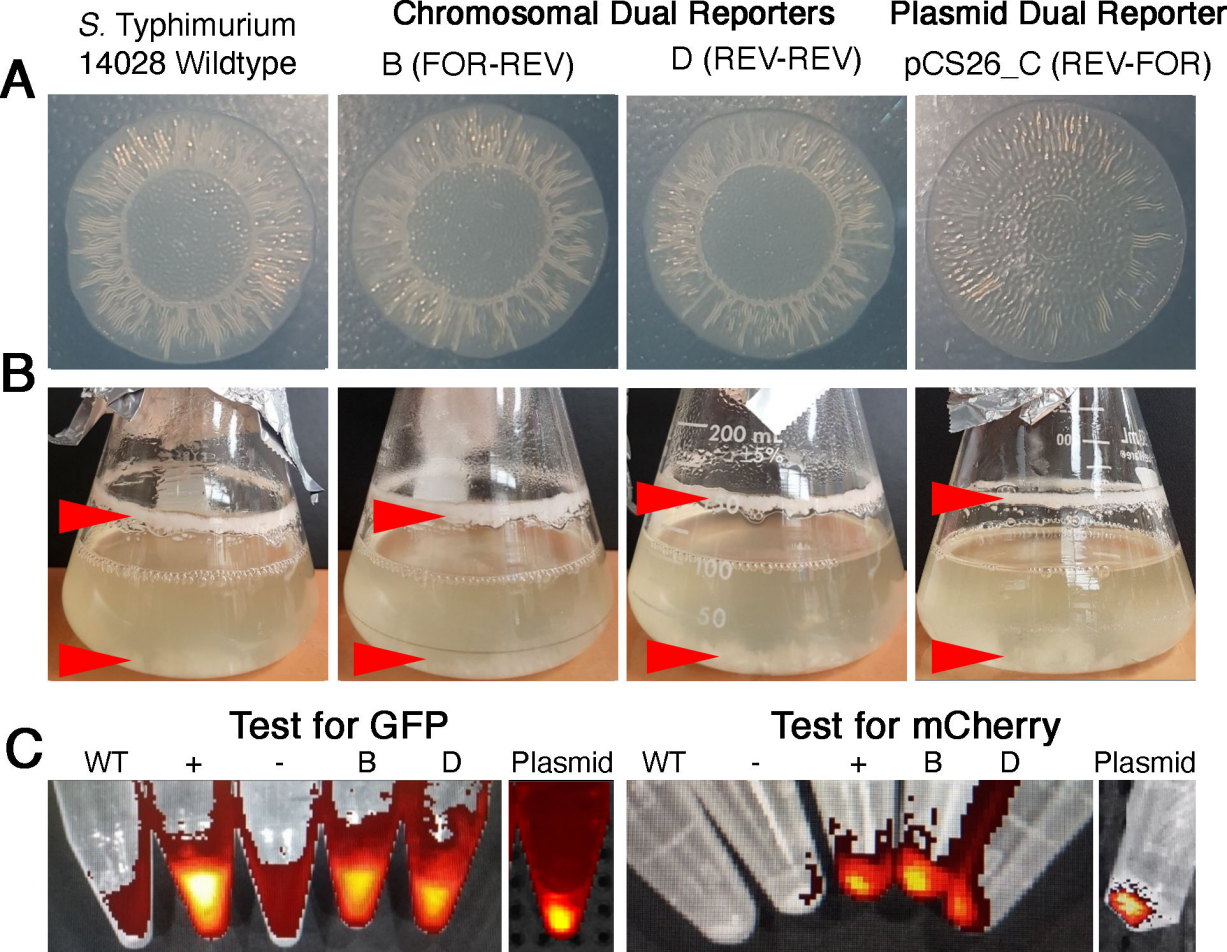
Screening for biofilm phenotypes and fluorophore expression in *S*. Typhimurium dual reporter strains. *S*. Typhimurium 14028 dual reporter strains “B” and “D” and plasmid dual reporter strain “C” were compared to the 14028 wild-type strain and evaluated for their ability to form the red, dry, and rough (rdar) morphotype, which presents as the formation of concentric rings and a wrinkled appearance on the surface of microcolonies (**A**) and for the presence of multicellular, biofilm aggregates and planktonic cells in liquid cultures grown under biofilm-inducing conditions. (**B**) Biofilm aggregates formed at the air–liquid interface and bottom of each flask culture are indicated with red triangles. (**C**) From the flask cultures, biofilm aggregates were tested for production of GFP (500 nm excitation; 540 nm emission), and planktonic cell fractions were tested for production of mCherry (570 nm excitation; 620 nm emission) using an IVIS Spectrum CT *in vivo* imaging system. *S*. Typhimurium *csgB::GFP* and *prgH::mCherry* single reporter strains were also included and tested as positive (+) and negative (−) controls. The bright yellow color indicates production of the appropriate fluorophore.

Each of the *S*. Typhimurium dual reporter strains was screened to ensure that the biofilm development pathways were still intact. *S*. Typhimurium reporter strains B and D both formed the characteristic rough biofilm colony morphology on 1% tryptone agar (Fig. 3A) and differentiated into biofilm aggregates and single-cell fractions during growth in the biofilm flask model (Fig. 3B). When the biofilm and planktonic cell fractions in the culture were harvested, biofilm cells were positive for GFP fluorescence, and planktonic cells were positive for mCherry fluorescence (Fig. 3C). The intensity of the fluorescence signals was comparable to the previously generated single reporter strains for biofilm and SPI-1 T3SS. These results indicated that the dual reporters could track *csgB*-positive and *prgH*-positive cell types without impairing biofilm formation. The plasmid-based dual reporter (pCS26_C; Fig. 2) also displayed similar biofilm phenotypes and fluorescence profiles (Fig. 3). We also screened more than 60 individual colonies for *S*. Typhimurium dual reporter strains A and C (Fig. 2A), but none of them grew well in the presence of kanamycin, and they failed to produce the expected rough biofilm colony morphology (Fig. S3). Therefore, reporter strains A and C were excluded from further analysis.

### Chromosomally-integrated dual reporter strains have relatively uniform gene expression

**Fig 4:**
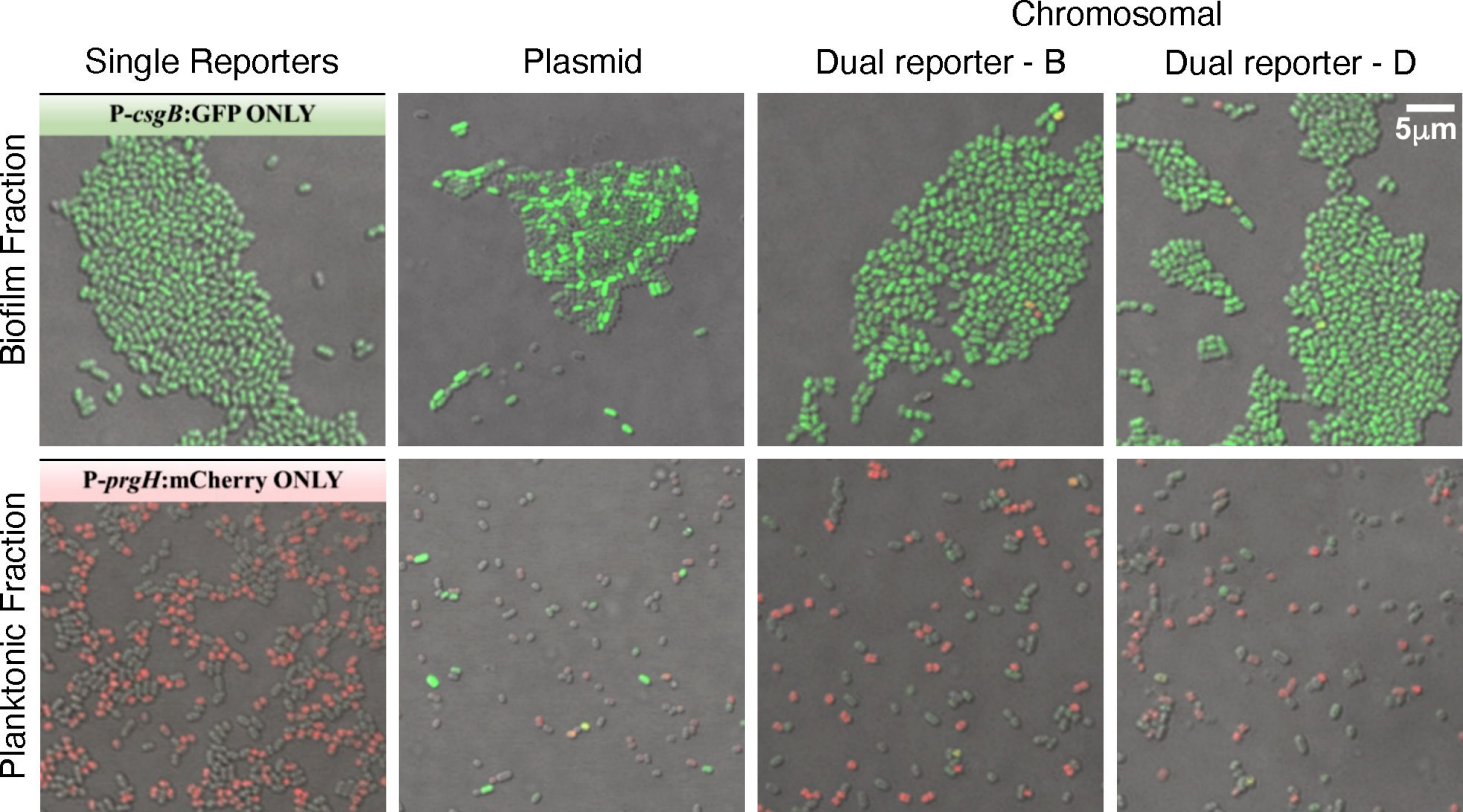
Fluorescence analysis of *S*. Typhimurium 14028 reporter strains at the single-cell level. Representative confocal microscopy images are shown for cells from the biofilm and planktonic fractions isolated from *S*. Typhimurium 14028 liquid cultures grown under biofilm-inducing conditions. *S*. Typhimurium 14028 chromosomal single reporter strains (i.e., P-*csgB*:GFP [top] and P-*prgH*:mCherry [bottom]) were compared to the plasmid dual reporter “C” strain and chromosomal dual reporter strains “B” and “D.” Green cells represent curli-positive cells, and red cells represent SPI-1 T3SS-positive cells. Yellow or orange cells represent cells expressing both GFP and mCherry. GFP, mCherry, and DIC images were captured sequentially, aligned, and merged using Leica software. Images for the plasmid strain were adjusted to avoid oversaturation of GFP-positive cells.

We compared the fluorescence of biofilm and single-cell populations between the chromosomally integrated and plasmid-based dual reporter strains at the single-cell level. Strains were grown in an *in vitro* flask model for biofilm development, and the biofilm and planktonic cell populations were processed and visualized using a Leica SP5 confocal microscope. Both B and D dual reporter strains showed relatively uniform fluorescence that was comparable to the chromosomally integrated *csgB::GFP* and *prgH::mCherry* single reporter strains (Fig. 4). We noticed a significant number of non-fluorescent cells in the planktonic cell populations, but the distribution was similar to the *prgH::mCherry* single reporter strain. In contrast, the plasmid-based dual reporter strain C showed a much wider range of fluorescent intensity between individual cells (probably reflective of variable copy number), with GFP-positive cells often appearing highly saturated. This created challenges in capturing weaker GFP-positive cells and mCherry-positive cells. Based on the increased variation in gene expression, we discontinued using this plasmid-based reporter strain.

### Quantification of four cell populations in biofilm flask cultures using *S*. Typhimurium dual reporter strain “D”

**Fig 5:**
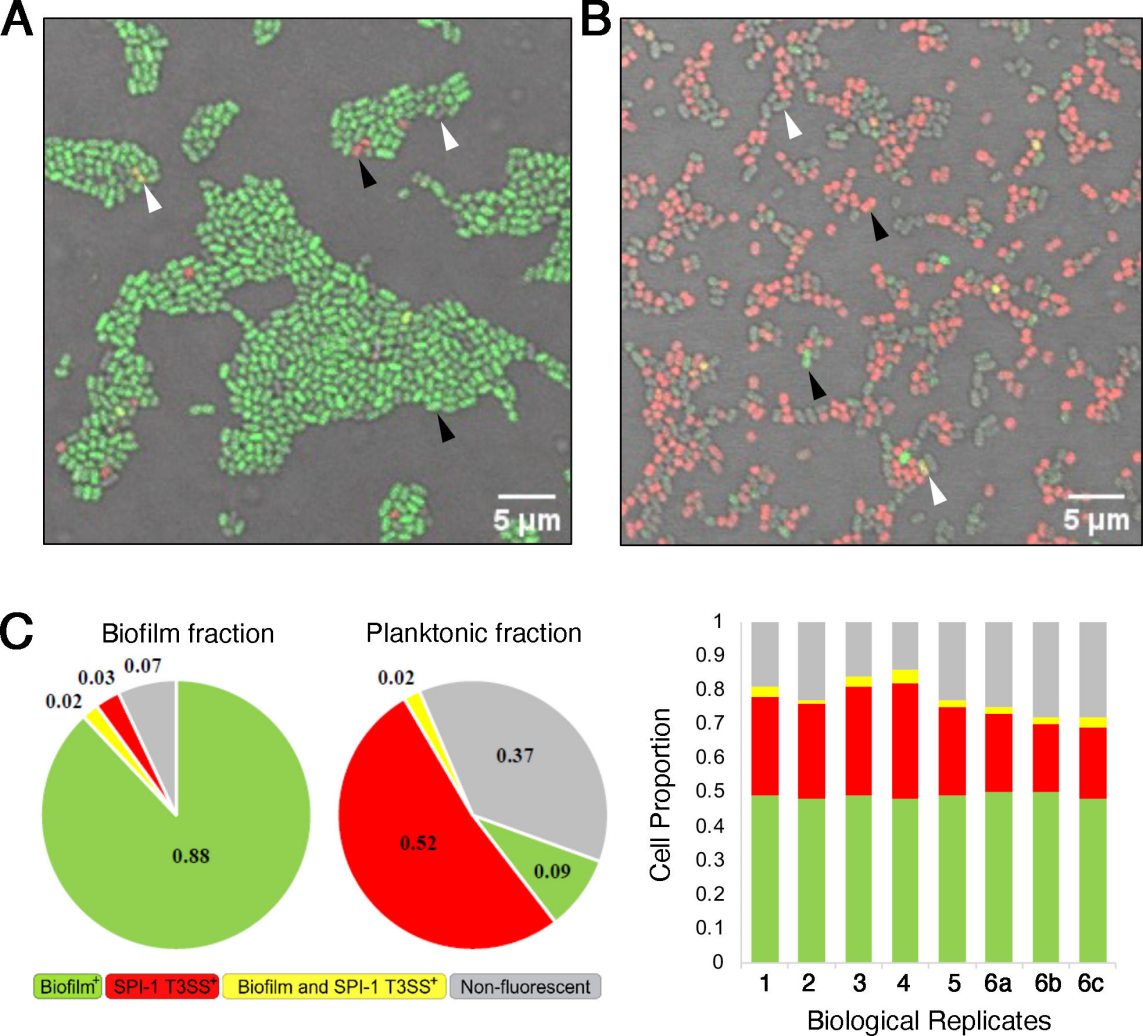
Quantifying the levels of GFP and mCherry expression in biofilm and planktonic fractions from *S*. Typhimurium 14028 dual reporter strain “D.” Representative confocal microscopy images of a biofilm sample (**A**) and planktonic sample (**B**) isolated from liquid cultures of *S*. Typhimurium 14028 dual reporter strain “D.” Cells were harvested, placed on agarose pad slides, and images were captured on a Leica SP8 confocal microscope using a 63× oil immersion lens. GFP, mCherry, and DIC images were captured sequentially, aligned, and merged using Leica software. Black arrows point to GFP-positive (curli+) and mCherry-positive (SPI-1 T3SS+) cells, and white arrows point to cells that are either non-fluorescent or positive for both fluorophores. (**C**) Pie charts represent the average proportion value for cell types present in the biofilm and planktonic cell fractions: Biofilm^+^ (GFP-positive), SPI-1 T3SS^+^ (mCherry-positive), Biofilm^+^ and SPI-1 T3SS^+^ (GFP− and mCherry-positive), and non-fluorescent. The total proportion of each cell type was quantified across six biological replicate flask cultures, including three technical replicates of flask #6 (6a, 6b, and 6c), to demonstrate the variability.

We quantified the proportion of different cell types in the dual reporter strain D. Six biological and three technical replicate flask cultures were grown, and the biofilm and single-cell populations were separated and analyzed by confocal microscopy. Both biofilm and planktonic cell fractions contained four distinct cell types: biofilm^+^ cells (GFP^+^/mCherry^−^) and SPI-1 T3SS^+^ cells (GFP^−^/mCherry^+^) (Fig. 5A and B; black arrows), as well as dual-positive cells (GFP^+^/mCherry^+^) and non-fluorescent cells (GFP^−^/mCherry^−^) (Fig. 5A and B; white arrows). The biofilm fraction consisted of 88% biofilm^+^ cells, with 7% non-fluorescent, 3% SPI-1 T3SS^+^ cells, and 2% double-positive cells (Fig. 5C); these unexpected cell types were observed to often be located at the edges of individual biofilm aggregates (Fig. 5A). The single-cell population consisted primarily of SPI-1 T3SS-positive (52%) and non-fluorescent cells (37%) with 9% biofilm cells and 2% double-positive cells (Fig. 5C). When data from both biofilm and single-cell fractions were combined, approximately half of all cells analyzed were biofilm-positive (0.48), followed by SPI-1 T3SS^+^ cells (0.27), non-fluorescent cells (0.23), and double-positive cells (0.02). The proportion of biofilm^+^ cells was the most consistent across replicates, ranging from 0.48 to 0.50, whereas SPI-1 T3SS^+^ cells varied from 0.20 to 0.34 and non-fluorescent cells varied from 0.14 to 0.28. The non-fluorescent cells were present in both biofilm and single-cell samples, but were significantly more abundant in the single-cell fraction. Even though the double-positive cells were rarer, they were consistently observed in all cultures, ranging from 0.01 to 0.04 (Fig. 5C).

### The *S*. Typhimurium 14028 dual reporter strain can infect *C. elegans* and proliferate in the intestine

**Fig 6:**
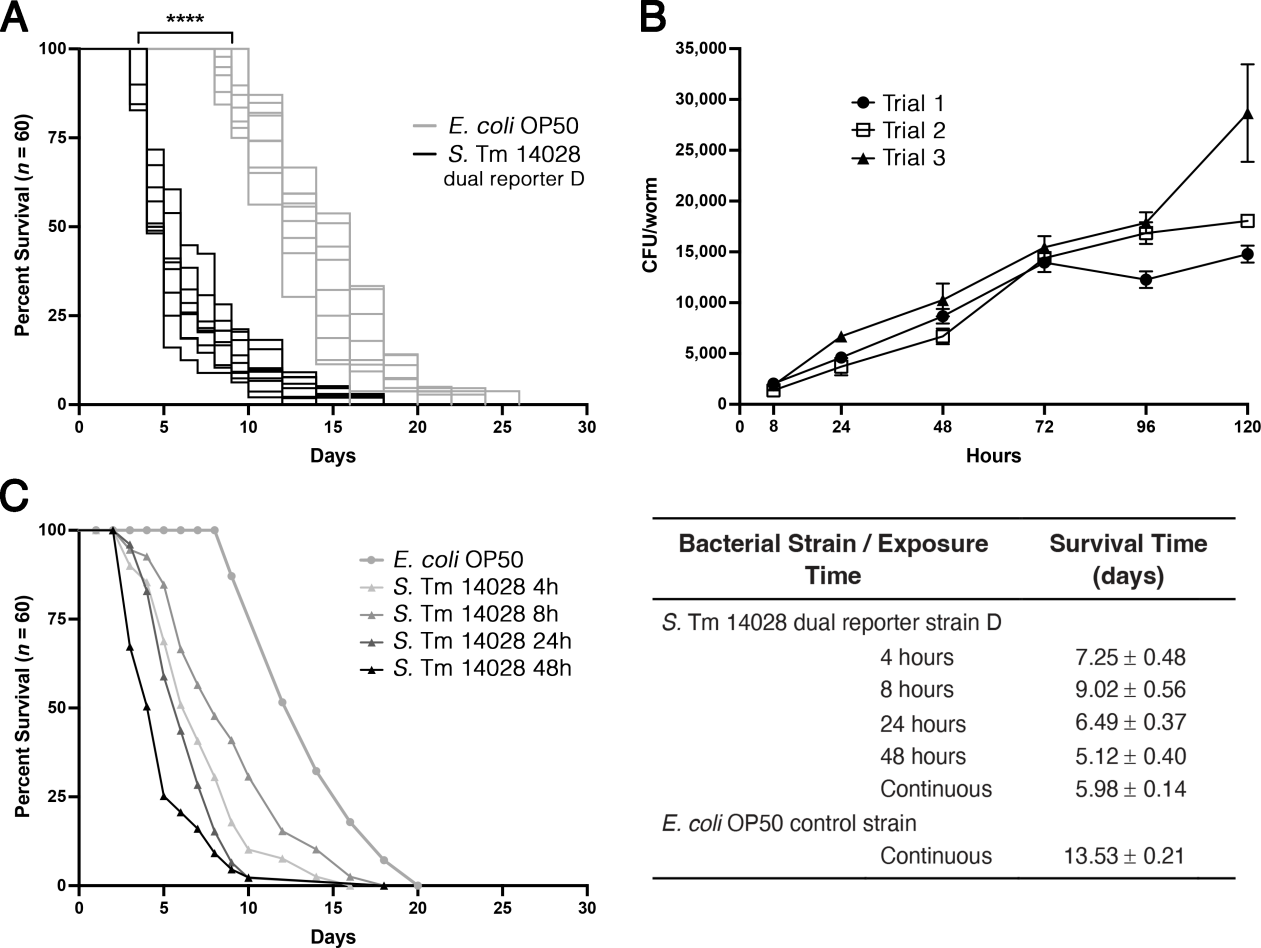
Infection of *C. elegans* with *S*. Typhimurium dual reporter strain “D.” (**A**) Survival of adult wild-type N2 worms grown with the food source as *S*. Typhimurium 14028 dual reporter strain “D” (black curves) or *E. coli* OP50 (grey curves). Three independent trials were completed with 3 replicates per trial and *n* = 60 worms in each replicate; *P* < 0.0001. (**B**) The number of *S*. Typhimurium cells (CFU) inside worms was determined for the first 5 days of infection. Data are shown from three independent trials, and error bars show the standard error of the mean. (**C**) Survival of adult N2 worms after exposure to *S*. Typhimurium 14028 dual reporter strain “D” for 4, 8, 24, or 48 h (grey and black lines with triangles) and subsequent feeding on *E. coli* OP50 or continuous feeding on *E. coli* OP50 (grey line with circles). The mean survival values on the right (± the standard error) were calculated from the survival curves using SPSS software. The continuous exposure values correspond to *n* = 540 worms, whereas the shorter exposure times correspond to *n* = 60 worms.

*C. elegans* has been used previously as an infection model for non-typhoidal *Salmonella* serovars (33, 34). These worms are a relatively simple model organism, and the transparency of the intestine allows for tracking of fluorescent cells. We performed a lifespan experiment where worms were continuously exposed to the *S*. Typhimurium dual reporter strain D starting from the first day of adulthood. Worms exposed to *S*. Typhimurium had a significantly shortened lifespan compared to those continuously feeding on *E. coli* OP50 (Fig. 6A). In each trial, worms exposed to *Salmonella* appeared to be sick and fragile after 3 days, and mortality reached 100% by day 18. In contrast, worms grown on *E. coli* OP50 remained healthy until at least day 10, with complete worm death by day 26. We measured the bacterial load of *S*. Typhimurium in the *C. elegans* intestine. At 8 h post-exposure, up to 2,000 bacterial cells were recovered, and this number increased nearly 10-fold by day 5 (Fig. 6B). From 8 to 72 h post-exposure, the mean CFU per worm data were relatively consistent, whereas the 96 and 120 h time points had more variability (Fig. 6B). We attributed this variability to worms becoming fragile at later time points and rupturing during the washing and handling steps. We conducted a second lifespan experiment where worms were exposed to *S*. Typhimurium for defined timeframes before being transferred back to plates colonized by *E. coli* OP50. The survival curves of worms exposed to *S*. Typhimurium for 4, 8, 24, and 48 h were similar to each other, displaying the same shortened lifespan that ended by 18 days (Fig. 6C). The mean survival of the *Salmonella-*infected worms was calculated and ranged from 5 days to 9 days, all significantly less than the ~13-day mean survival time of worms continuously feeding on *E. coli* (Fig. 6C). In addition to the decreased lifespan, there were morphological changes in the worms infected with *S*. Typhimurium. After 24 h, the worms feeding on *Salmonella* or *E. coli* appeared similar in appearance, with worms displaying smooth body surfaces with minimal foregut (pharynx) swelling (Fig. S4). The worms feeding on *E. coli* OP50 still retained a smooth body at 96 h (Fig. S4B), whereas the worms infected with *S*. Typhimurium appeared wrinkled, and the pharynx was swollen and distorted (Fig. S4D). These results confirmed that the *S*. Typhimurium dual reporter strain D can infect, colonize, and induce progressive tissue damage in *C. elegans*, likely due to the disruption of epithelial integrity.

### *S*. Typhimurium displays dynamic gene expression inside the *C. elegans* intestine

**Fig 7:**
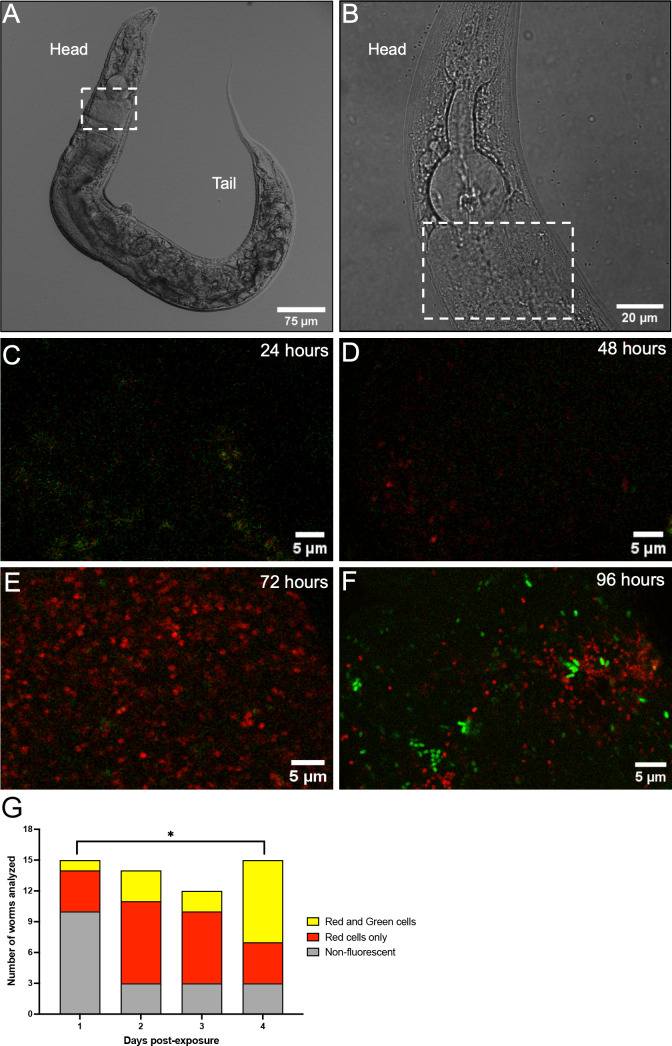
Evidence of *S*. Typhimurium population splitting inside the *C. elegans* intestine. Confocal microscopy images were generated from the intestinal region of *C. elegans* after colonization with *S*. Typhimurium 14028 dual reporter strain D. DIC images are shown for two adult nematodes viewed with a 20× objective lens (**A**) or a 63× oil immersion lens (**B**), each with a white dashed line box to show the region of the worm that was captured at later time points. Representative images of the *C. elegans* intestine are shown at 24 h (**C**), 48 h (**D**), 72 h (**E**), and 96 h (**F**) post-infection with *S*. Typhimurium. Worms were immobilized on an agarose pad and were visualized on a Leica SP8 confocal microscope. (**G**) Worms at each time point (*n* = 12–15) were collected and characterized as having only non-fluorescent cells present, only red cells (mCherry-positive) present, or both red (mCherry-positive) and green (GFP-positive) cells present. The difference in fluorescence distribution between Day 1 and Day 4 was significant (*P* = 0.009).

The *S*. Typhimurium cell populations proved to be highly dynamic in the *C. elegans* intestine over time. Analysis of *S*. Typhimurium cells on NGM agar prior to *C. elegans* exposure indicated that most cells (80%) were in the non-fluorescent state (Fig. S5). After 24–48 h of exposure, *Salmonella* cells were primarily non-fluorescent or could not be visualized (Fig. 7C and D). Some of these worms showed intestinal swelling and dark puncta in the intestine, which may represent non-fluorescent *Salmonella* cells (Fig. S6). By 72 h of exposure, *Salmonella* cells consistently were SPI-1 T3SS positive (Fig. 7E). By 96 h of exposure, both *Salmonella* SPI-1 T3SS^+^ cells and biofilm^+^ cells were visible in the worm intestine (Fig. 7F). Our experimental setup did not allow us to track *S*. Typhimurium within the same individual worms over time; therefore, we imaged multiple, infected worms at each time point to generate a qualitative summary of *S*. Typhimurium gene expression. The whole intestine of each worm was imaged, and the worms were scored by what *S*. Typhimurium cell type appeared to be the most abundant. Despite variations between individual worms, there was a clear trend with *S*. Typhimurium cells starting as primarily non-fluorescent on Day 1 to primarily mCherry-positive (SPI-1 T3SS^+^) on Day 2 and Day 3, and to the majority of worms containing distinct groups of cells that were either mCherry-positive (SPI-1 T3SS^+^) or GFP-positive (biofilm^+^) on Day 4 (Fig. 7G). The difference between Day 1 and Day 4 was statistically significant. In some worms, the transition of *S*. Typhimurium cells from non-fluorescent to SPI-1 T3SS^+^, and from primarily SPI-1 T3SS^+^ to both SPI-1 T3SS^+^ cells and biofilm^+^ cells, occurred at earlier time points. Some worms contained fluorescent *Salmonella* throughout the intestine, while some only contained fluorescent *Salmonella* in the region behind the pharynx and at the posterior end of the intestine. The ratio of SPI-1 T3SS^+^ cells and biofilm^+^ cells inside the worms imaged at Day 4 was more difficult to quantify, and it is possible that non-fluorescent cells were also present, as we observed significant intestinal swelling and dark puncta visible that were not fluorescent (Fig. S6).

### Differentiation into biofilm^+^ and SPI-1 T3SS^+^ cell populations is a common trait in non-typhoidal *Salmonella* strains

**Fig 8:**
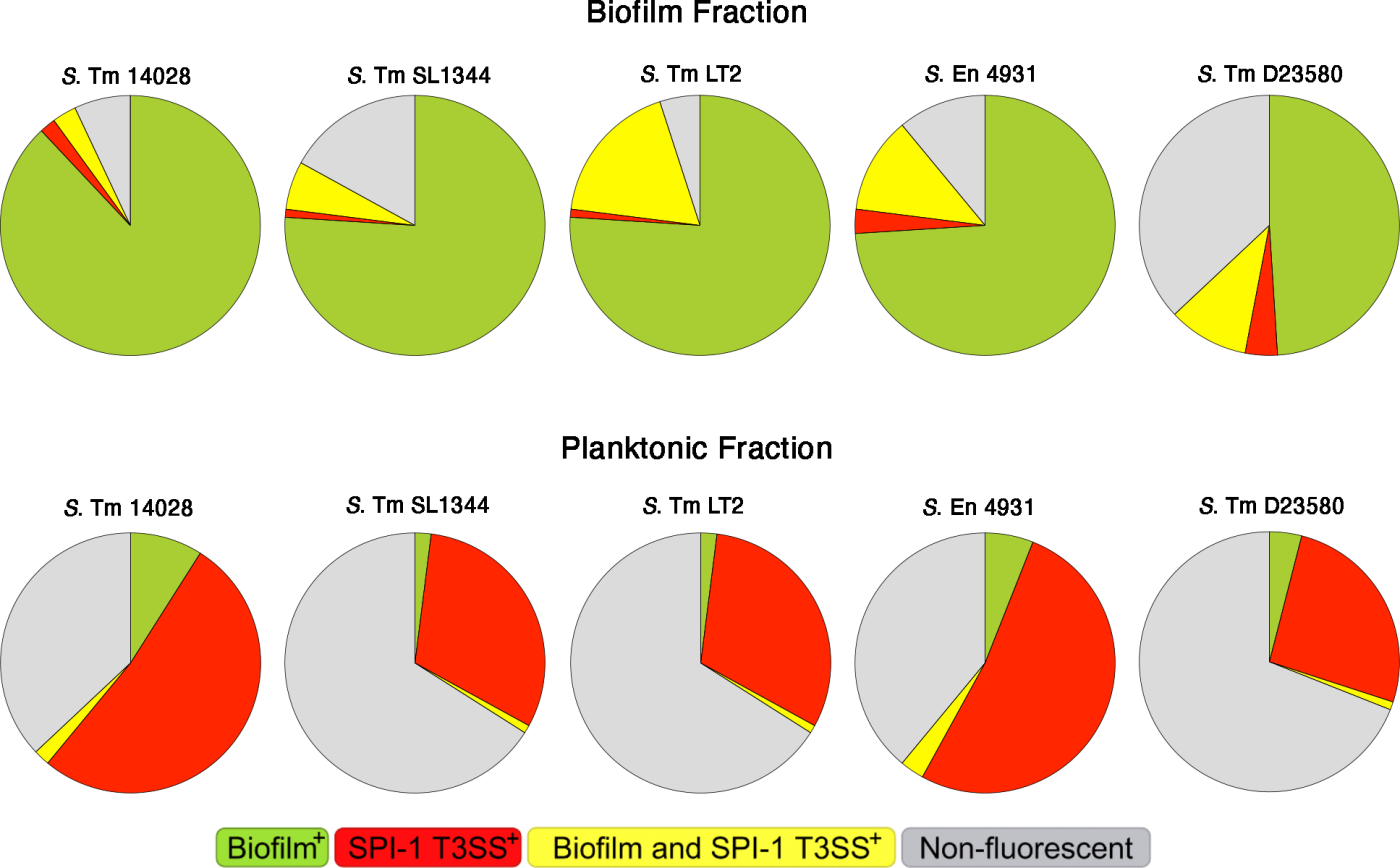
Quantifying cell types using the dual reporter construct in diverse NTS strains. Dual reporter construct “D” was mobilized from *S*. Typhimurium 14028 using P22 phage and inserted into the chromosomes of *S*. Typhimurium SL1344, *S*. Typhimurium LT2, *S*. Typhimurium D23580, and *Salmonella* serovar Enteritidis ATCC 4931. These dual reporter strains were evaluated by growing cultures under biofilm-inducing conditions, separating the biofilm and planktonic fractions, and analyzing the proportions of different cell types by confocal microscopy. The pie charts show the proportion of each cell type that was present; a total of 20,000 cells were analyzed from each new dual reporter strain. Pie chart data from *S*. Typhimurium 14028 was repeated from Fig. 5.

To determine whether the cellular differentiation observed in *S*. Typhimurium 14028 is a common trait with other NTS; we moved the *csgB::GFP* /*prgH::mCherry* dual reporter construct into three additional strains that cause gastroenteritis (i.e., *S*. Typhimurium LT2, *S*. Typhimurium SL1344, and S. Enteritidis 4931) and another strain, *S*. Typhimurium D23580, that causes a more invasive type of *Salmonella* infection in humans (35, 36). When each new dual reporter strain was grown in biofilm flasks, the same four cell types were observed. Biofilm gene expression was the most conserved, with the exception of D23580, which had fewer biofilm-positive cells (Fig. 8). All strains had a small proportion of SPI-1 T3SS-positive cells present in the biofilm fraction and a relatively high percentage of double-positive cells. In the planktonic cell fractions, all strains again showed all four cell types, but for three strains (SL1344, LT2, and D23580), the non-fluorescent cell population was the most abundant (Fig. 8). *S*. Enteritidis 4931 had the most similar distribution of fluorescent cell types as compared to *S*. Typhimurium 14028. We are unsure of the significance of the differences between strains in the proportions of the cell types observed. However, it was clear from this analysis that CsgD/SPI-1 T3SS cell differentiation is a common feature of NTS strains.

## DISCUSSION

Bet-hedging is thought to be an evolutionary strategy for bacteria and even higher organisms (37, 38) to survive unpredictable environments (1, 3, 39, 40). We have proposed that bet-hedging is a mechanism to split populations of *Salmonella enterica* cells into virulence-expressing cells that can initiate infection and persistence-oriented cells that can withstand environmental stresses (7). This study represents the first time that this process has been studied at the single-cell level. We examined in detail how the bistability of CsgD and biofilms may be intertwined with SPI-1 T3SS bistability (41, 42). Bistable networks are used by bacteria to divide labor among specialized cell types to efficiently complete a complex task and save energy, or as a survival strategy when dealing with uncertain conditions (3, 5, 6, 43). We previously analyzed the genetic and phenotypic differences between biofilm aggregates and planktonic cells in a flask model of biofilm development and showed that more than 35% of the genes in the *S*. Typhimurium genome were differentially expressed (19). When competitive infections in C57BL/6 mice were performed, the planktonic cells were significantly more virulent than biofilm aggregates. It should be noted that *S. enterica* serovar Typhi strains form biofilms on human gallstones in a process independent of CsgD (44), but we are not sure if bistable expression occurs under these conditions. In this study, we showed that the *S*. Typhimurium biofilm population has some SPI-1 T3SS-expressing cells present, and the planktonic cell population has some biofilm cells present as well as a large proportion of SPI-1 T3SS non-expressing cells. A comparison between a pure population of biofilm cells and a pure population of SPI-1 T3SS-expressing cells would likely reveal even greater differences than what we measured before. The interpretation of what the different *Salmonella* cell populations are biologically adapted for is hard to quantify, but it indicates that a genetic program of differentiation exists. A similar concept has been established for *Vibrio cholerae*, in which bistable control of virulence gene expression is thought to contribute to future transmission success (9). Vibrio biofilm clumps present in rice water stools are thought to allow for long-term survival in the environment, such as on copepods in seawater (45, 46), but their role is complex as they are also hyper-infectious (47, 48). The transmission cycle of *Salmonella* is not as well understood as with *V. cholerae*, so perhaps there are more nuances in the roles of both biofilm and planktonic cells than what we have proposed.

We wanted to establish the dynamics of CsgD and SPI-1 gene expression inside a living host. Although the virulence of *S*. Typhimurium has traditionally been studied in susceptible mouse models (i.e., Nramp-deficient), there are limitations, as the infection more closely resembles typhoid fever in humans than it does gastroenteritis (49). In addition, the murine intestinal tract is 30 cm long, and large portions of tissue are autofluorescent, making it difficult to track fluorescent bacterial cells. *C. elegans* represents a much simpler host with the advantages of high throughput and ease of tracking cells inside the transparent intestine (33, 34). We showed that *Salmonella* ingested by worms led to colonization and alteration of the worm appearance, coupled with significantly reduced lifespan. Fluorescent imaging revealed temporal restructuring of *S*. Typhimurium cell types within the intestine, as cells entered as primarily non-fluorescent, shifted to SPI-1 T3SS-expressing cells by Days 1–3, and a final shift to both SPI-1 T3SS-expressing and biofilm-expressing cells being present. This temporal pattern aligns with previous studies showing that SPI-1 expression emerges after ~48 h of infection (50) and that large biofilm aggregates appear by 6 days (51). The purpose of *S*. Typhimurium biofilm formation inside *C. elegans* is not fully understood. Desai et al. proposed that biofilm formation prolonged worm lifespan, leading to more persistent infections (51), whereas Thiers et al. (52) found that biofilm formation did not influence worm survival or MAPK intestinal signaling. We observed that biofilm-positive cells expanded as the worm population declined and wondered if the final shift was a response to tissue destruction, as the appearance of biofilm cells often matched with impending worm death. We will be able to address this question more directly by tracking individual infected worms over time. *Salmonella* is known to use cues such as inflammation, nutrient shifts, and the host physiological decline as a trigger for the sequential activation of transcriptional regulatory pathways (53, 54). Early-stage expansion of non-fluorescent cells might be advantageous for initial colonization; however, it should be noted that we could not visualize these cells directly. The mid-stage expansion of SPI-1 T3SS^+^ cells confirms the requirement of invasion-associated factors that disrupt epithelial barriers and withstand host defenses (13, 55). The emergence of biofilm-positive cells and shift toward persistence could be driven by immune evasion, nutrient depletion, or reduced oxygenation. The role of cellulose in this process is complex; cellulose is produced as part of CsgD-mediated biofilm formation via activation of diguanylate cyclase AdrA (17), but it can also be produced independently of CsgD, where it seems to have an anti-virulence role (22). It has been shown that both SPI-1 T3SS and non-expressing cells are needed for optimal virulence in mouse models, but we do not know whether this also applies to *C. elegans*. Nevertheless, our results showed that the *S*. Typhimurium cell population changed dynamically during infection.

The presence of four distinct cell types in *S*. Typhimurium biofilm flask cultures revealed significant heterogeneity within the cell population. Quantification of ~80,000 cells from multiple replicates revealed that these populations are reproducible, indicating that their formation is controlled by regulatory mechanisms, and potentially bet-hedging, as observed in other phenotypic diversification processes (40, 56). It is possible that multiple bistable genetic networks are at play to generate CsgD-ON and CsgD-OFF populations, as well as SPI-1-ON and SPI-1-OFF populations. We and others have shown that the signaling for biofilm formation involves nutrient scarcity or depletion (57, 58), but we are unsure how this applies to SPI-1 T3SS activation since the biofilm conditions (i.e., low osmolarity, 28°C) do not match standard conditions for SPI-1 activation (i.e., high osmolarity, 37°C) (59, 60). Bistable expression of SPI-1 and the formation of SPI-1 negative cells is thought to be crucial for stabilizing *Salmonella* populations during an infection (41, 42, 61, 62). At present, we do not know whether the non-fluorescent cells in the biofilm population are the same as the non-fluorescent cells in the planktonic population or if these are yet again different cell types. Neutral or metabolically less committed cell populations have been identified in previous single-cell and transcriptomic studies on heterogeneous *Salmonella* cultures, where non-growing or slow-growing subpopulations have been observed (56, 63, 64). Low-activity cells are thought to represent growth-maximizing subpopulations or could provide a flexible reservoir ready to transition into a more specialized state, which fits well with current models of division of labor and bet-hedging in *Salmonella* populations (19, 42). It was observed previously that in an SPI-1 bistable cell population there are many more SPI-1 OFF cells than SPI-1 ON cells due to their faster growth (42). Non-fluorescent cells in our study were equal to or fewer in number than SPI-1 T3SS^+^ cells, but this could simply reflect different culture conditions. For double-positive cells, biofilm genes and SPI-1 genes being expressed at the same time seems unlikely, given the differences in functioning of these two phenotypes and the significant energetic expense required; therefore, we predict that the double-positive cells represent a transitional state. It is possible that the SsrA/B two-component system regulates the switch between *Salmonella* invasion and persistence (51). Phosphorylated SsrB can bind to regulatory regions to repress SPI-1 transcription (65), whereas unphosphorylated SsrB can bind to other regions to upregulate *csgD* expression (66). Although double-positive cell types were not observed in the worm intestine, they might have been obscured by naturally occurring autofluorescent granules. Understanding how host cues, regulatory circuits, and stress responses influence the transition between *S*. Typhimurium cell types can be studied in the future using single-cell transcriptomics and real-time imaging.

We have shown that non-typhoidal *Salmonella* maintain a diverse population of coexisting cell types, presumably balancing growth, virulence, and persistence in their unique ways. All NTS strains that we tested diversified into the same four cell subpopulations in the biofilm flask model, although their relative abundance varied. The differences in proportion could be the result of different evolutionary histories for each strain. For example, we and others have shown that the invasive non-typhoidal strain (D23580) has reduced biofilm formation and decreased CsgD activity (20, 22, 67–69). The reproducibility of the cell types across strains argues that the observed transitions are not biological noise but rather are regulated and evolved strategies that *Salmonella* relies on. The SPI-1-positive and biofilm-positive cells represent two high-investment ecologically important strategies, geared toward invasion and long-term persistence. The presence of double-positive cells indicates that transitions between states might occur more frequently than what classical models of SPI-1 or CsgD bistability would predict. Overall, these findings depict a population-level bet-hedging strategy in which distinct cell types share the workload to maximize survival and resilience under fluctuating environmental conditions.

## MATERIALS AND METHODS

### Bacterial strains

All bacterial and nematode strains used in this study are listed in Table S1. All cloning steps were performed in *E. coli* DH10B or *E. coli* CC118(*λ pir*) (pUC18R6K-mini-Tn7T-PacI). For general growth, *E. coli* strains were inoculated from frozen stocks and grown overnight at 37°C in Luria-Bertani broth (LB) supplemented with antibiotics (i.e., 10 mg/mL chloramphenicol, 50 mg/mL kanamycin, and 100 mg/mL ampicillin).

### Gene expression analysis of key virulence promoters in *S*. Typhimurium 14028

DNA regions were PCR amplified from *S*. Typhimurium 14028 using Phusion high-fidelity DNA polymerase (New England BioLabs, Ipswich, MA, USA), following the manufacturer’s instructions, with primers invF1 and invF2 (*invF* promoter); primers hilA2 and hilA3 (*hilA* promoter); primers hilD1 and hilD2 (*hilD/prgH* promoters) (Table S2). PCR products were purified, digested with *Xho*I and *Bam*HI, and ligated using T4 DNA ligase (New England BioLabs, Ipswich, MA, USA) into pCS26 (*hilA*, *invF,* and *hilD*) or pU220 (*prgH*) digested with the same enzymes. PCR screening with primers pZE05 and pZE06 (Table S2), followed by DNA sequencing, was used to verify the successful fusion of promoter regions to *luxCDABE,* and the plasmids were transformed into *S*. Typhimurium 14028. *csgD and csgB* luciferase fusion plasmids have been previously described (13).

For analysis of gene expression, overnight cultures of *S*. Typhimurium luciferase reporter strains were diluted 1 in 600 into wells of black, clear-bottom 96-well plates (9520 Costar; Corning Life Sciences, Tewksbury, MA, USA) containing 150 μL of 1% tryptone broth supplemented with 50 μg/mL kanamycin (Kan). Prior to growth, media were supplemented with 10% of effluent from a continuous culture chemostat seeded with human fecal samples, or with 10% of a chemostat media-only control (70). Wells were overlaid with 50 μL of mineral oil to minimize evaporation, and cultures were assayed for absorbance (600 nm, 0.1 s) and luminescence (1 s; in counts per second [CPS]) every 30 min during growth at 28°C with agitation using a Victor X3 multilabel plate reader (Perkin-Elmer, Waltham, MA, USA).

### Generation of *Salmonella* SPI-1 and dual reporter strains

The *csgB::GFP*/*prgH::mCherry* dual reporter constructs were synthesized as five DNA fragments (IDT Canada), and Golden Gate assembly (New England Biolabs, # E1601S) was used to construct them into the pGGA recipient plasmid (Fig. S7). The dual reporter constructs were cut from pGGA using *Pac*I and were ligated into *Pac*I-digested pUC18R6K-miniTn7T-PacI. 300 ng of each pUC18R6K plasmid was electroporated into *S*. Typhimurium 14028 *ΔcsgD*-competent cells containing the pHSG415-*tnsABCD* helper plasmid, and chromosomal integration was performed using Tn7 transposition (31). Plasmid dual reporter strain “C” was constructed by ligating *Pac*I-digested fragment “C” into *Pac*I-digested pCS26 (32) and transforming *S*. Typhimurium 14028. *S*. Typhimurium 14028 *ΔcsgD* strains containing the dual reporter constructs were grown overnight at 37°C in LB broth supplemented with kanamycin (50 µg/mL). A P22 phage donor lysate was generated and passed through a 0.22 µM filter into clean glass vials (71). Aliquots of 100 µL of overnight cultures of recipient *Salmonella* strains (*S*. Typhimurium 14028, *S*. Typhimurium SL1344, *S*. Typhimurium LT2, *S*. Enteritidis 4931, and *S*. Typhimurium D23580) were mixed with 10 µL of donor lysate, and the transduced cells were selected by growing on LB agar supplemented with kanamycin (50 µg/mL) and EGTA (5 mM). To obtain phage-free reporter cells, potential transductant colonies were grown on Green agar media, and freezer stocks were made. The DNA sequences of all *S. enterica* dual reporter strains were confirmed by DNA sequencing. Detailed information on generating a SPI-I T3SS single reporter strain and whole-genome sequencing is shown in Methods S1 and S2.

### Screening *S*. Typhimurium 14028 dual reporter strains for biofilm phenotypes

Potential dual reporter strains of *S*. Typhimurium 14028 were grown overnight at 37°C in LB broth supplemented with kanamycin (50 µg/mL). Cultures were diluted to an OD_600_ of 1.0, and 4 µL was spotted onto 1% tryptone media containing 1.5% agar and 50 µg/mL kanamycin. Cells were grown at 28°C for 40 h–72 h, and colony morphology was compared to wild-type 14028. To test strains for cellular differentiation into biofilm aggregates and planktonic cells, overnight cultures were normalized to an OD_600_ of 1.0, and 1 mL aliquots were inoculated into 100 mL of 1% tryptone broth supplemented with 50 µg/mL kanamycin. Flask cultures were incubated at 28°C for 18 h in a water bath with agitation (200 rpm). Biofilm and planktonic cell fractions were separated by low-speed centrifugation (210 × *g*; 2 min), as previously described (19). Information on imaging of planktonic and biofilm details is shown in Methods S3.

### Fluorescence microscopy analysis of *S*. Typhimurium 14028 dual reporter strains

To visualize individual cells of *S*. Typhimurium 14028 dual reporter strain D, biofilm flask cultures were prepared as previously described (19). Detailed information on sample preparation and analysis using ImageJ is found in Methods S4 and S5.

### *C. elegans* infections and survival assays

#### Maintenance of *C. elegans* and *S*. Typhimurium infection experiments

Wild-type *C. elegans* strain N2 worms were maintained on Nematode Growth Media (NGM) that was seeded with *E. coli* strain OP50 and incubated at 23°C. Standard protocols were followed as previously described (72). To assess the survival of *C. elegans* infected with *S*. Typhimurium 14028 dual reporter D, wild-type N2 worms were synchronized using a standard bleaching protocol (73) and grown to the first day of adulthood on two separate NGM plates seeded with *E. coli* OP50 (stock plates). Sixty day one adult worms were moved to NGM plates seeded with the *S*. Typhimurium 14028 dual reporter strain or NGM plates seeded with *E. coli* OP50. Worms were transferred to fresh plates and scored daily until day 10, after which worms were no longer transferred to new plates and were scored every second day. For testing how different exposure times affected survival, worms were exposed to the *S*. Typhimurium 14028 dual reporter strain D for 4, 8, 24, and 48 h before transferring back to fresh *E. coli* OP50-NGM plates for the remainder of the experiment. Worms were considered dead if they did not respond to light touch. Worms that crawled up the sides of the plates and died, or any that were accidentally killed were censored from the data set (Table S3). Quantification of intestinal bacterial load per worm, cell-type enumeration from NGM agar plates, and microscopy of individual infected worms were performed as described in Methods S6 to S8.

#### Statistical analysis

All statistical tests were performed using SPSS, GraphPad Prism, and/or Microsoft Excel. For *C. elegans* lifespan experiments, mean worm survival and standard error were determined in SPSS using a Kaplan–Meier survival analysis. The mean time until death between worm groups was tested by removing censored worms and using a Mann–Whitney U test. For *Salmonella* CFU per worm experiments, the standard error of the mean CFU/worm triplicate plating was calculated using Microsoft Excel.

## Supplementary Material

Reviewer comments

## Data Availability

All data collected and used to generate the figures are available upon request.

## SUPPLEMENTAL MATERIAL

Supplemental material msystems.00177-26-s0001.pdf Figure S1 to S7, Tables S1 to S3, and Methods S1 to S8.

OPEN PEER REVIEW reviewer-comments.pdf An accounting of the reviewer comments and feedback.

ASM does not own the copyrights to Supplemental Material that may be linked to, or accessed through, an article. The authors have granted ASM a non-exclusive, world-wide license to publish the Supplemental Material files. Please contact the corresponding author directly for reuse.
