# Supplementary material for: Exploring bet-hedging in Salmonella enterica serovar Typhimurium with a dual reporter strain
Source: mSystems. 2026 May 18;11(6):e00177-26. doi: 10.1128/msystems.00177-26 (PMC13289073; doi:10.1128/msystems.00177-26)
Supplement: Supplemental material — Figure S1 to S7, Tables S1 to S3, and Methods S1 to S8. [file msystems.00177-26-s0001.pdf]

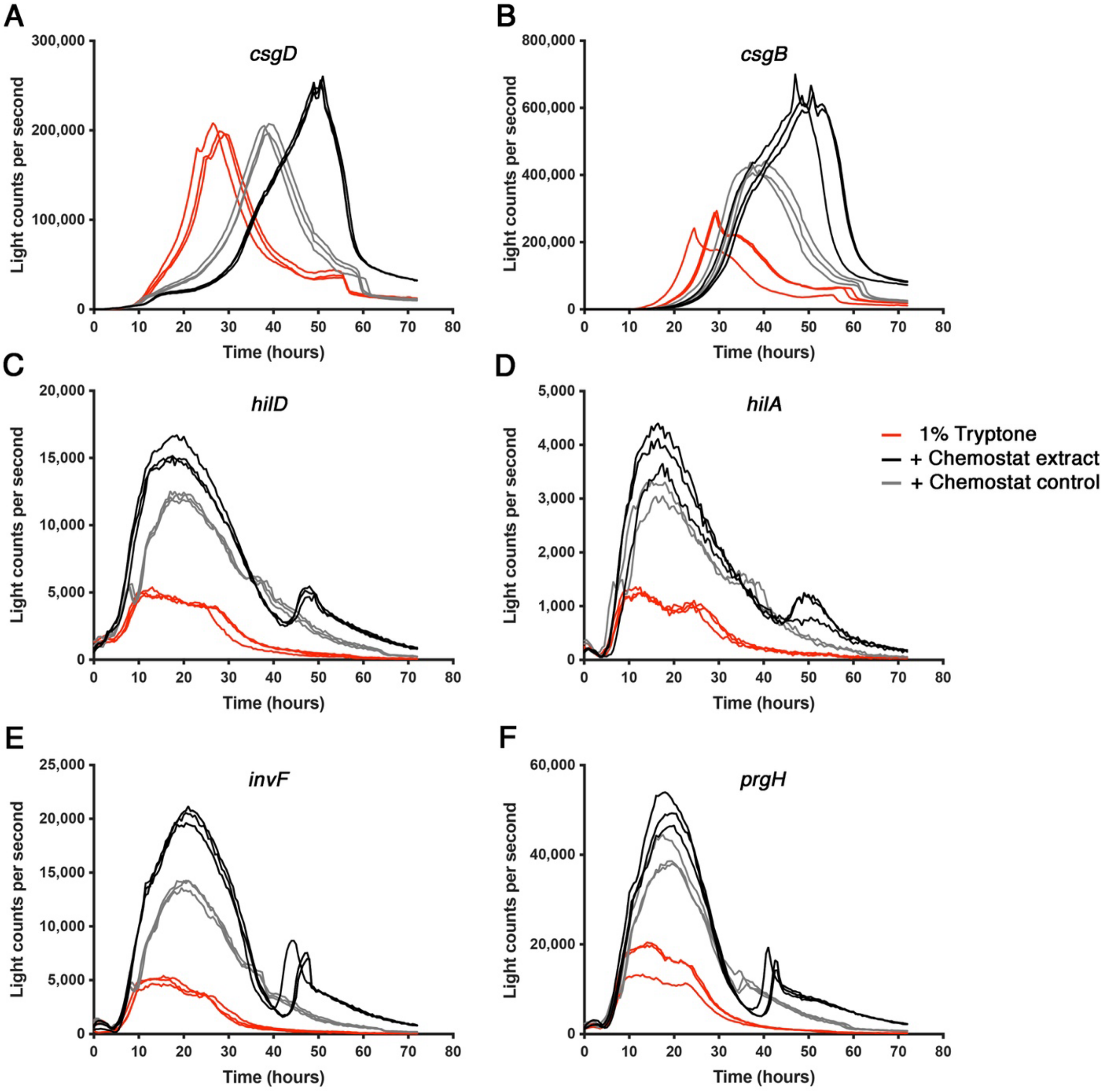

Fig. S1. Response of the *S.* Typhimurium CsgD and SPI-1 T3SS regulatory networks to human fecal extract. Expression from the *csgD* (A), *csgB* (B), *hilD* (C), *hilA* (D), *invF* (E) and *prgH* (F) promoters was measured in *S*. Typhimurium 14028 during growth at 28ºC in 1% tryptone media or media supplemented with 10% of an extract from a human fecal continuous culture chemostat (chemostat extract) or 10% of a chemostat media control (both courtesy of Dr. Emma Allen Vercoe, University of Guelph), For each graph, luminescence (light counts per second) was plotted as a function of time with 3-5 curves in each condition representing different biological replicates. Luminescence and optical density measurements for growth were recorded every 30 min for a total of 72 h.

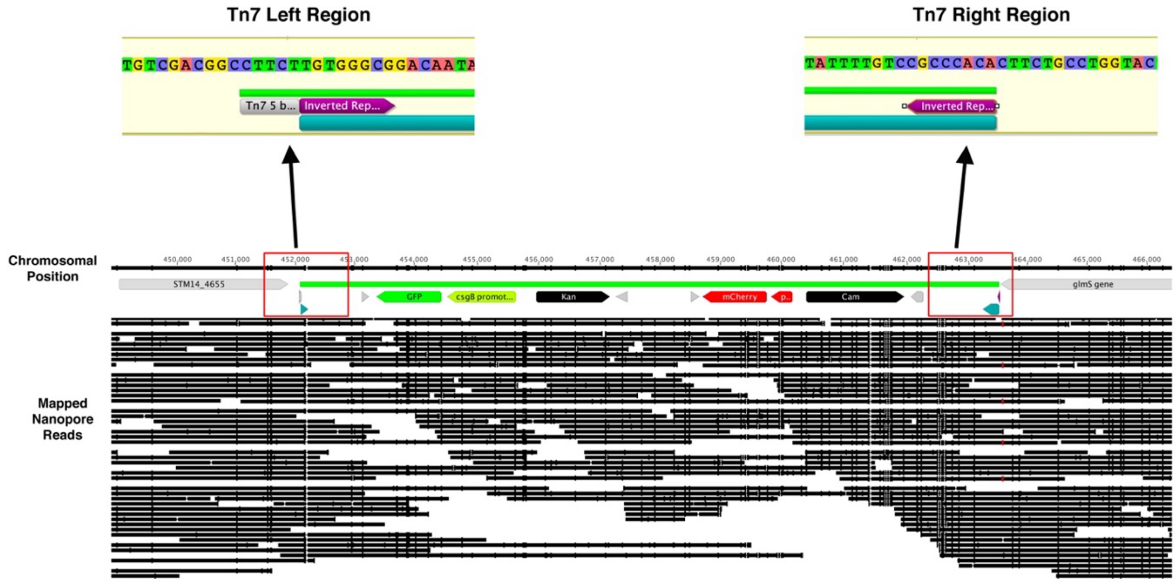

Fig. S2. Identification of 5 base pair signature of Tn7 transposition in *S*. Typhimurium 14028 dual reporter "D".
Schematic showing a region of the Nanopore Whole Genome sequencing mapped reads of *S*. Typhimurium 14028 with the dual reporter construct "D" inserted into the chromosome. The reporter construct was inserted into the attTn7 site downstream of *glmS* (grey bar on the right). The nucleotide sequence of the outer regions of the Tn7 left and right ends is magnified to show a 5 base pair duplicated sequence (CTTCT; grey box) that was identified outside the inverted repeats of the Tn7 left and right ends (purple regions).

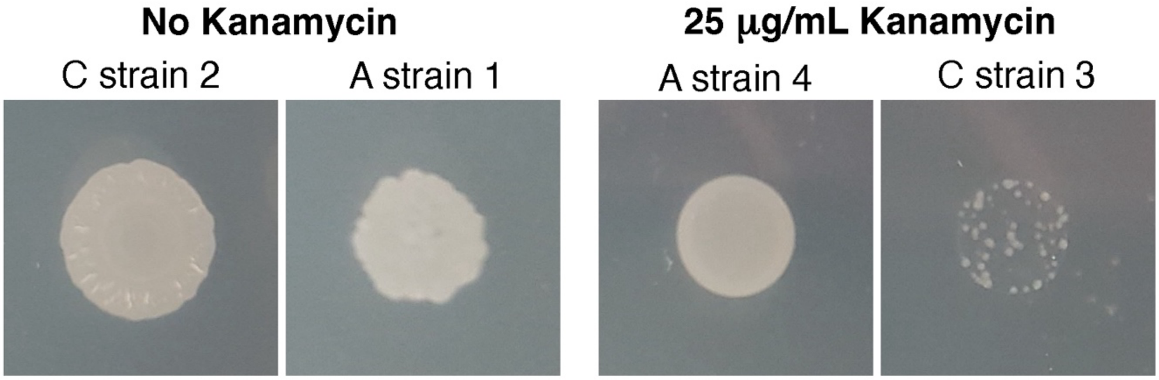

Fig. S3. *S*. Typhimurium 14028 dual reporter strains "A" and "C" did not produce the expected biofilm colony morphology. Images showing examples of the colony morphologies produced by two potential *S*. Typhimurium 14028 chromosomal dual reporter strains when tested for rdar biofilm colony morphology. 4 µL aliquots of culture (~7.7 x 10^7 cells) were spotted on 1% Tryptone agar and the plates were incubated for 40 h at 28°C.

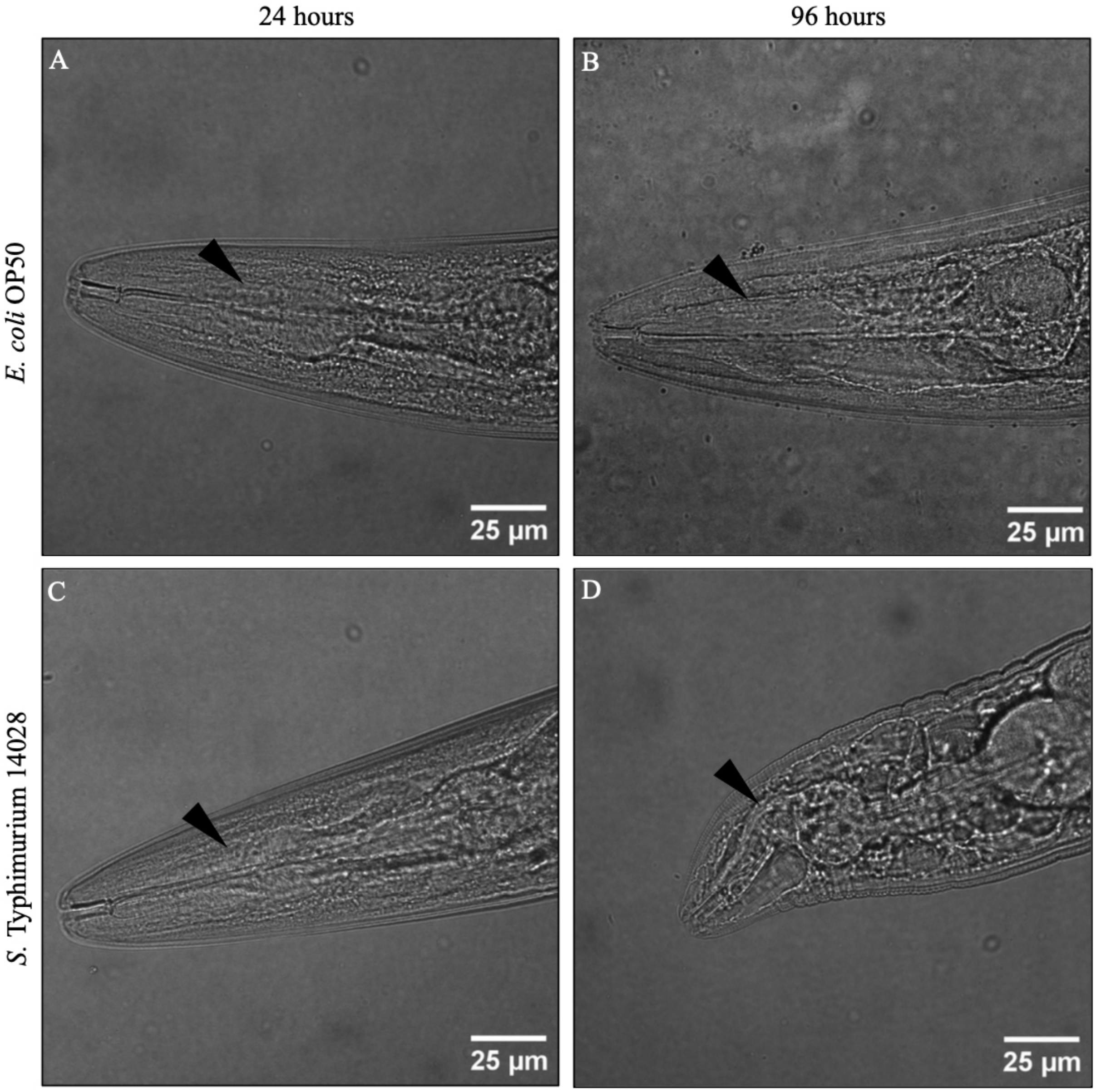

Fig. S4. Changes to the body of *C. elegans* during growth with *E. coli* or *S*. Typhimurium as the food source. DIC confocal microscopy images showing the foregut of 4 different adult N2 nematodes eating either *E. coli* OP50 (A and B) or *S*. Typhimurium 14028 dual reporter strain D (C and D) for 24 h or 96 h. The pharynx is indicated with a black triangle.

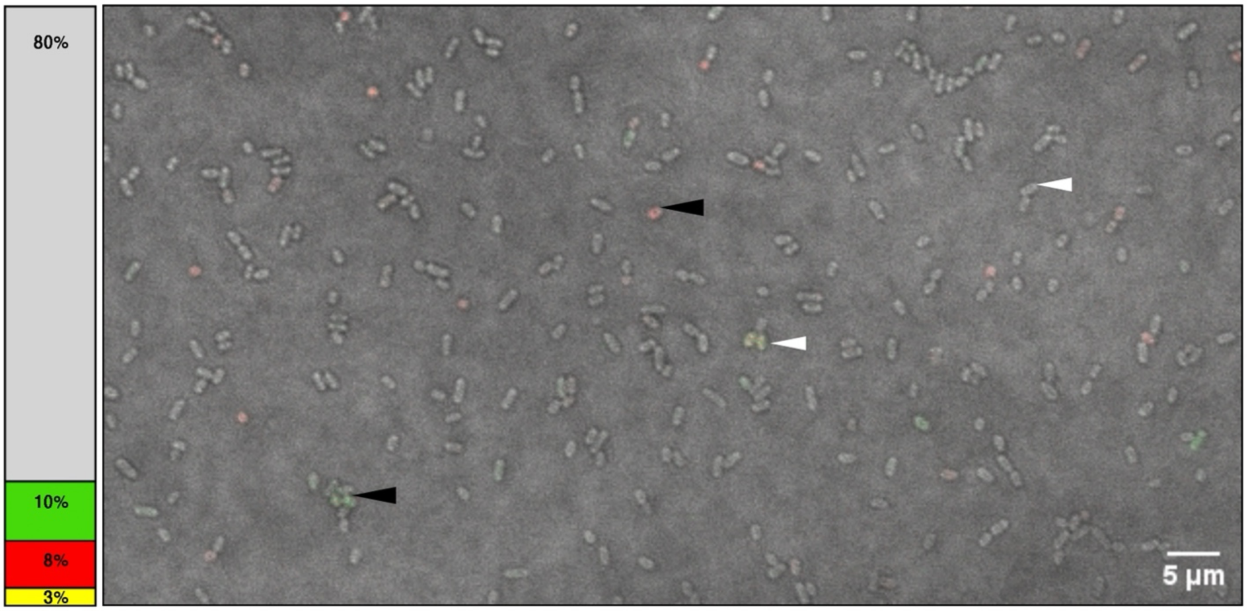

Fig. S5. *S.* Typhimurium 14028 dual reporter D cells are primarily non-fluorescent prior to ingestion by *C. elegans*. Cells were scraped off an NGM agar plate after growth at room temperature for 24 h and visualized on a Leica SP8 confocal microscope using a 63X oil immersion objective lens. Black arrows point to GFP-positive (curli+) and mCherry-positive (SPI-1 T3SS+) cells, and white arrows point to cells that are either non-fluorescent or positive for both fluorophores. Bar on left shows the proportions of each cell type, based on analysis of 10,000 cells. NGM plates were prepared this way for *S.* Typhimurium infection experiments.

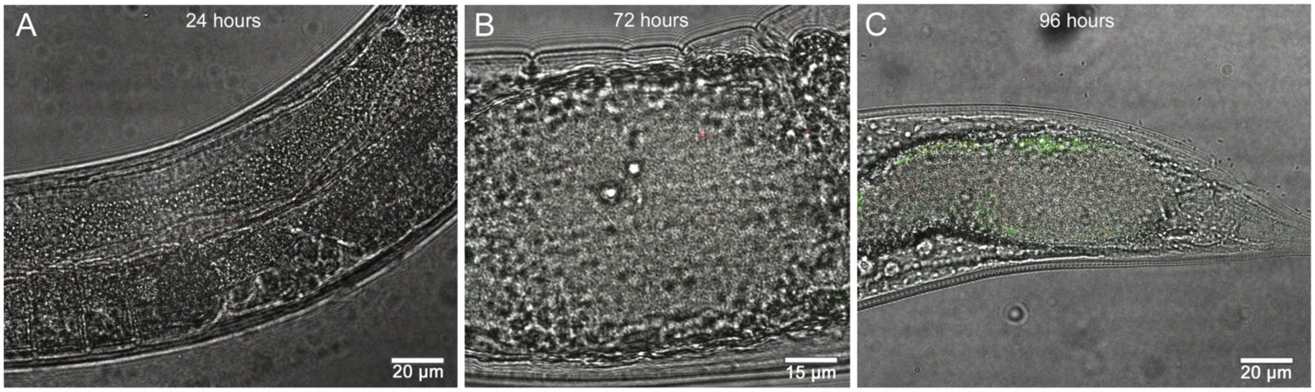

Fig. S6. Inflamed *C. elegans* intestine containing *S.* Typhimurium cells. Representative confocal microscopy images showing the intestine of 3 different adult N2 nematodes after infection with *S*. Typhimurium 14028 dual reporter strain D for 24h (A), 72 h (B) or 96 h (C). Note the dark puncta visible inside each worm, regions that we think could contain non-fluorescent *S.* Typhimurium cells.

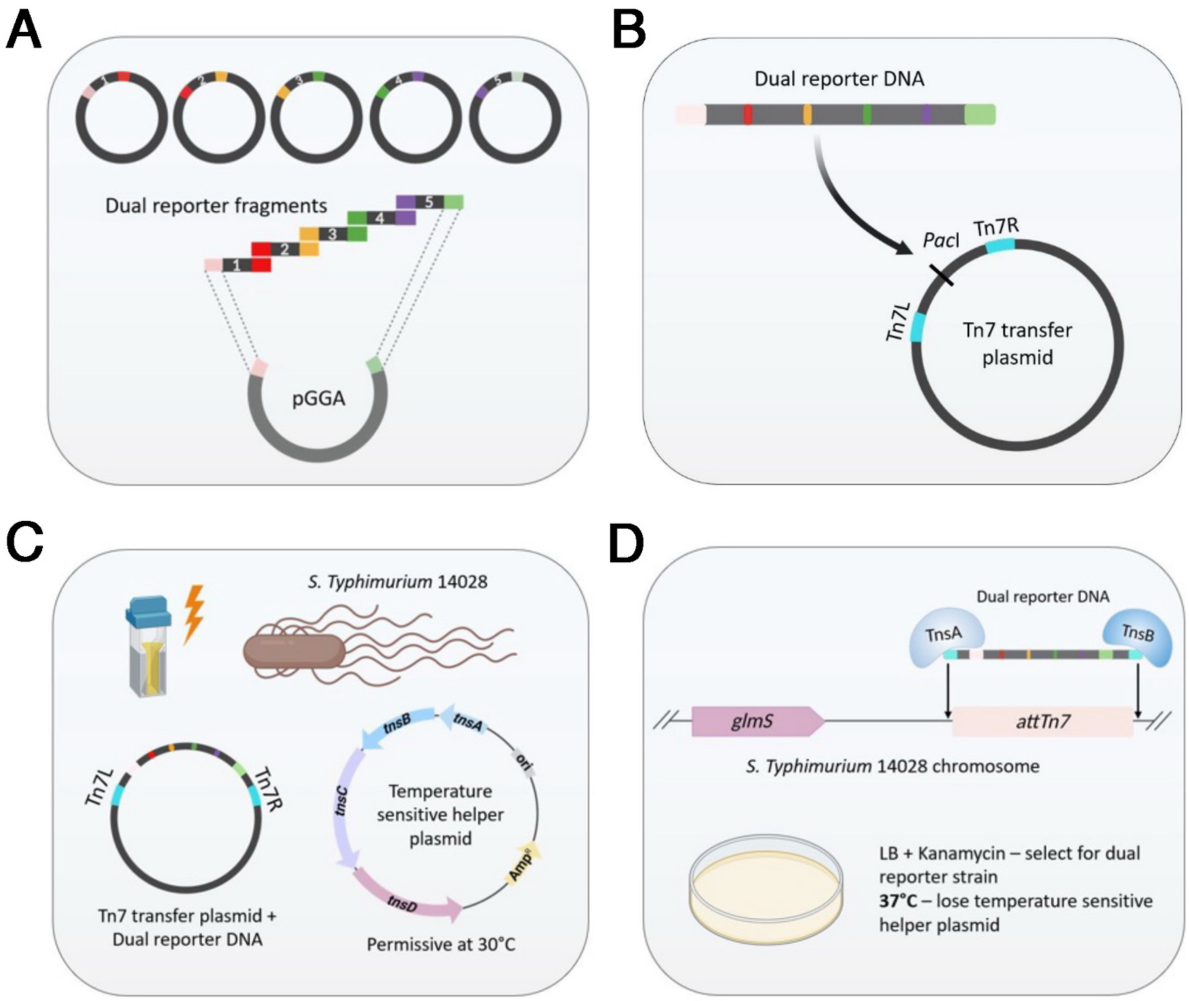

Fig. S7. Dual Reporter Strain Assembly Workflow. Schematic showing the steps taken to build the *S.* Typhimurium dual reporter strains. A) Dual reporter DNA fragments were designed using Geneious R9 software (geneious.com) and synthesized into plasmids by IDT. The DNA fragments were assembled in order using Golden Gate Cloning into the vector plasmid pGGA. B) The dual reporter construct was ligated into the *Pac*I site between the Tn7 left and right ends of a Tn7 transfer plasmid (pUC18R6K-mini-Tn7T-PacI; Shivak et al., 2016). C) The Tn7 plasmid containing the dual reporter construct was electroporated into *S.* Typhimurium 14028 cells that contain a temperature sensitive helper plasmid (Amp^R) which encodes the Tn7 transposition machinery proteins (pHSG415-*tnsABCD*; Shivak et al., 2016). Cells were incubated at the permissive temperature for 3 hours for recovery and for transposition to take place. D) During the incubation, TnsB binds to the Tn7 left and right ends and mobilizes the dual reporter DNA into the conserved *att*Tn7 site in the *S.* Typhimurium 14028 genome downstream from *glmS* in a specific orientation. Cells were plated onto selective agar and incubated at the non-permissive temperature to ensure loss of the helper plasmid. Colonies were screened for Kan^R and the dual reporter DNA was moved into a clean wildtype *S.* Typhimurium 14028 background using P22 bacteriophage. Figure was created using Biorender software.

Table S1. Bacterial and Nematode strains used in this study

**Strain**[a]	**Genotype**	**Source or Reference**
E. coli DH10B	F– *mcrA* Δ(*mrr-hsdRMS-mcrBC*) φ80*lacZ*ΔM15 Δ*lacX74 recA1 endA1 araD139* Δ (*ara-leu*)7697 *galU galK* λ– *rpsL*(Str^R) *nupG*	M. Surette (McMaster University)
E. coli CC118(*λpir*)		Herrero M., 1990
E. coli CC118(*λpir*)	pUC18R6K-mini-Tn7T-PacI	Shivak et al., 2016
S. Typhimurium ATCC 14028s	Wildtype	M. Surette (McMaster University)
Tn7 helper strain	*ΔcsgD* pHSG415-*tnsABCD*	Shivak et al., 2016
biofilm single reporter	*csgD::GFP* - Kan^R	MacKenzie et al., 2015
virulence single reporter	*prgH::mCherry* - Cam^R	This study
Plasmid dual reporter "C"	pCS26 – *csgB::GFP* Kan^R – Cam^R *prgH::mCherry*	This study
Dual reporter "B"	*GFP::csgB* – Kan^R - Cam^R *-prgH::mCherry*	This study
Dual reporter "D"	*GFP::csgB* – Kan^R – *mCherry::prgH* – Cam^R	This study
S. Typhimurium SL1344	Wildtype	W. Köster (University of Saskatchewan)
Dual reporter "D"	*GFP::csgB* – Kan^R – *mCherry::prgH* – Cam^R	This study
S. Typhimurium LT2	Wildtype	K. Sanderson (University of Calgary)
Dual reporter "D"	*GFP::csgB* – Kan^R – *mCherry::prgH* – Cam^R	This study
S. Typhimurium D23580	Wildtype	G. Dougan (Sanger Institute, UK)
Dual reporter "D"	*GFP::csgB* – Kan^R – *mCherry::prgH* – Cam^R	This study
S. Enteritidis 4931	Wildtype	D. Korber (University of Saskatchewan)
Dual reporter "D"	*GFP::csgB* – Kan^R – *mCherry::prgH* – Cam^R	This study
Caenorhabditis elegans N2	Wildtype	Caenorhabditis Genetics Center (CGC)

Reference

Herrero M, de Lorenzo V, Timmis KN. Transposon vectors containing non-antibiotic resistance selection markers for cloning and stable chromosomal insertion of foreign genes in gram-negative bacteria. J Bacteriol. 1990 Nov;172(11):6557-67. doi: 10.1128/jb.172.11.6557-6567.1990. PMID: 2172216; PMCID: PMC526845.

Table S2. Oligonucleotides used in this study.

Primer Name	Sequence (5'-3')[a]	Purpose
hilA2	GATCGGATCCGATAATAGTGTATTCTCTTAC	To amplify *hilA* promoter region from *S*. Typhimurium 14028
hilA3	GATCCTCGAGGATGATACTGCTCATAACCCT	
invF1	GATCCTCGAGTTAACTCCAACTACAGAAGAATGA	To amplify *invF* promoter region from *S*. Typhimurium 14028
invF2	GATCGGATCCAGTTGTCAGCACCAGTTAAAAATC	
hilD1	GATCCTCGAGATATACTGTTAGCGATGTC	To amplify the region with *hilD*/*prgH* promoters from *S.* Typhimurium 14028
hilD2	GATCGGATCCATTATCCCTTTGTTGATGT	
prgH1	GATCGGATCCTGATGTTTCCATATATACTG	To amplify *prgH* promoter region from *S.* Typhimurium 14028
prgH4	GATCCTCGAGCAATGGTCTGATTGTTACAC	
pZE05	CCAGCTGGCAATTCCGA	Used to verify promoter fusions to *luxCDABE*
pZE06	AATCATCACTTTCGGGAA	

[a] Nucleotide sequences corresponding to restriction enzyme sites are underlined.

Table S3. Summary of the Number of *C. elegans* Nematodes That Were Censored During the Lifespan Experiments

Bacterial strain / Trial / Replicate	Exposure Time	Worms Censored
E. coli OP50		
1 / 1	Constant	29
1 / 2	Constant	33
1/ 3	Constant	29
2 / 4	Constant	19
2 / 5	Constant	24
2 / 6	Constant	29
3 / 7	Constant	33
3 / 8	Constant	37
3 / 9	Constant	30
S. Typhimurium 14028		
1 / 1	Constant	5
1 / 2	Constant	6
1 / 3	Constant	6
2 / 4	Constant	16
2 / 5	Constant	16
2 / 6	Constant	15
3 / 7	Constant	7
3 / 8	Constant	6
3 / 9	Constant	7
4 / 1	4 hours	19
4 / 1	8 hours	15
4 / 1	24 hours	14
4 / 1	48 hours	12

* Worm censorship means that worms were not accounted for or died due to external factors. This could be worms that attempted to leave the agar surface and were found dried on the walls of the petri dish, or by accidental killing during manual transfer of worms to fresh agar plates.

SUPPLEMENTARY METHODS

S1: Generating a SPI-1 T3SS single reporter strain in *S*. Typhimurium 14028.

The *prgH* promoter region was PCR-amplified from *S*. Typhimurium 14028 using PCR primers prgH1 and prgH4 (Table S2). The PCR product was purified, digested with *Xho*I and *Bam*HI, and ligated into *Xho*I/*Bam*HI-digested pCS26-Cm (Shivak et al., 2016). The *prgH::mCherry* construct was cut out of this plasmid with *Pac*I and ligated into *Pac*I-digested pUC19R6KminiTn7T-PacI. Chromosomal integration of the *prgH::mCherry* construct into *S*. Typhimurium *ΔcsgD* was performed using an established Tn7 procedure (Shivak et al., 2016). P22 phage transduction was used to move the construct into the chromosome of wild-type *S*. Typhimurium 14028. Genome sequencing of the *S*. Typhimurium 14028 *prgH::mCherry* single reporter strain was performed on an Illumina MiSeq instrument, using a 2 x 300 bp flowcell and libraries produced with v3 chemistry. Whole genome assembly was performed using Geneious R11 software (Biomatters Inc.), using *S*. Typhimurium 14028 as the reference strain. The *csgB::GFP* biofilm single reporter strain was previously described (MacKenzie et al., 2015).

S2: Confirmation of dual reporter strains using Whole genome sequencing.

The final *S*. Typhimurium 14028 dual reporter strains were confirmed by whole genome sequencing performed in-house; DNA was purified using a GenElute Bacterial Genomic DNA Kit (Sigma-Aldrich; #NA2110), libraries were built using the Oxford Nanopore Ligation sequencing kit (SQK-LSK109), and sequencing was performed on the MinION Mk1C instrument, using R 9.4.1 flow cells. Genome assembly was performed using Geneious R11 software (Biomatters Inc.), mapping to *S*. Typhimurium 14028 as a reference genome. For *S*. Typhimurium LT2, SL1344, D23589 and *S*. Enteritidis 4931 dual reporter strains, Bacterial Genome Sequencing was performed by Plasmidsaurus using Oxford Nanopore Technology with custom analysis and annotation.

Importantly, a 5bp repeated sequence that is characteristic of Tn7 transposition (Figure S2; Waddell and Craig, 1989) was present in all dual reporter strains.

S3: Biofilm and planktonic cells fluorescence imaging.

Cell samples were prepared from cultures of an in vitro flask model of biofilm development. The supernatant was removed and planktonic cells were sedimented by centrifugation (10,400 × *g*; 2 min). Samples of biofilm cells, after removal of the supernatant, were transferred into 1.5 mL tubes using a sterile plastic loop. Cell fractions were evaluated for GFP fluorescence (500 nm excitation, 540 nm emission) and mCherry fluorescence (570 nm excitation, 620 nm emission) using an IVIS Lumina II Whole Animal Imager (Revvity). Cell fractions from biofilm flask cultures of wildtype *S.* Typhimurium 14028 (non-fluorescent), and *csgB::GFP* and *prgH::mCherry* single reporter strains were included as controls.

S4: Sample preparation for Fluorescence Microscopy.

After incubation, the flask culture was distributed into 15 mL centrifuge tubes, and the biofilm and planktonic cell fractions were separated by low-speed centrifugation (210 × *g*; 2 min). To ensure that any biofilm aggregates were separated from the planktonic cells, the tubes were centrifuged again (210 × *g*; 2 min). The top 15 mL of culture from each tube was transferred to a clean 50 mL centrifuge tube and used as the source of planktonic cells for microscopy. The remaining planktonic culture was filter sterilized (0.22 µM) to generate cell-free, nutrient depleted media. The biofilm cells were combined into a single 15 mL tube and washed 3 times with filter-sterilized nutrient depleted media until a clear supernatant was produced. Aliquots of ~30 mg of biofilm cells were added to 2 ml Eppendorf Safe lock tubes containing 1 mL of cell free spent media and a 5mm steel bead. Samples were homogenized using a MM100 mixer mill (Retsch) at 30 Hz for 5 min, diluted 1:2 in filter-sterilized nutrient depleted media, and 5 µL of the suspension was added

to an agarose pad slide. To generate slides of planktonic cells, the concentration of cells (CFU/mL) was estimated based on OD_{600} measurements and approximately 3.0 x 10^8 cells were added to an agarose pad slide. All slides were visualized with a 63X oil immersion objective lens with a 488/561/633 filter with the gain set at 965.0 V, and the frame average set at 8. Approximately 5,000 cells per slide were captured and 80,000 cells in total were quantified.

S5: Image processing and analysis pipeline using ImageJ.

All images were processed using the following ImageJ pipeline:

1. Raw images were cropped to create an evenly lit and level image for thresholding and to remove out of focus regions. For each slide, 10 cropped images containing approximately 500 cells each were analysed, yielding a total of 10,000 cells (5,000 planktonic + 5,000 biofilm) per flask culture.

2. The GFP, mCherry, and DIC raw images were merged to create a composite. All images were manually set to the same threshold value (130/255) and the total number of cells was counted using the Analyze Particles function and a size exclusion filter (0.18 µM – infinity).

3. All images were manually cleaned by splitting clearly merged ROIs or by adding any clearly missing ROIs with the ellipse drawing function. Manual ROIs were drawn using the fluorescent channels of the composite as a guideline to ensure the cell was properly captured.

4. The mean GFP and mCherry signal was measured for each cell using the multi-measure function in the ROI manager. To determine the lowest possible GFP and mCherry signal as a cut-off value, we measured the highest possible green fluorescence from the *prgH::mCherry* single reporter strain (MFI = ~ 4.0), and the highest possible red fluorescence from the *csgB::GFP* single reporter strain (MFI = ~ 11.0).

5. Cells with mCherry MFI greater than 11.0 were considered as SPI-1 T3SS positive and cells with GFP MFI greater than 4.0 were considered as biofilm positive. A background value of 1.0

MFI was subtracted from all images. All data were compiled, and the proportions of biofilm^{+} and SPI-1 T3SS^{+} cells were calculated using Microsoft Excel.

S6: Determining *S*. Typhimurium 14028 Intestinal Load per Worm Over Time.

We followed a previously described protocol (Palominos & Calixto, 2020) with some modifications. Wildtype N2 worms were synchronized and grown to adulthood on NGM seeded with *E. coli* OP50. Sixty adult worms were transferred to NGM plates seeded with the *S*. Typhimurium 14028 dual reporter strain D. At 8 h, 24 h, 48 h, 72 h, 96 h, and 120 h post-exposure, 30 worms were collected and series of washing steps were performed: three washes in M9 buffer + levamisole hydrochloride (25 mM), three washes with M9 buffer + levamisole hydrochloride (25 mM) + streptomycin (100 µg/mL) with a final 1 h exposure, and three final washes with M9 buffer + levamisole hydrochloride (25 mM). The cleaned worms were lysed using a sterile pestle (Fisher Scientific # 12-141-364) and resuspended in M9 buffer. Worm debris was sedimented by centrifugation (10,400 × *g*; 2 min) to create a stable pellet; the lysis and centrifugation steps were repeated until no intact worms remained, as visualized with a stereomicroscope. The resulting homogenate was serially diluted and inoculated in triplicate onto LB agar supplemented with kanamycin (50 µg/mL). The CFU per worm was calculated from three biological replicate experiments using the following equation:

$$CFU\ per\ worm = \frac{\left((Average\ \#\ colonies)\ x\ \left(\frac{1}{10^{Dilution\ Factor}}\right) x\ (Volume\ Plated)\right)}{30\ worms}$$

S7: Quantification of *S*. Typhimurium 14028 cell types from *C. elegans* agar plates.

A 200 µL aliquot of overnight culture of *S*. Typhimurium 14028 dual-reporter strain D was used to seed fresh NGM agar and plates were incubated at room temperature overnight. Bacterial cells were scraped from the agar surface with a sterile loop, transferred to a 1.5 mL microcentrifuge tube, and resuspended in filter sterilized nutrient depleted media. 5 µL of this cell suspension was

added to an agarose pad slide and imaged using a Leica SP8 confocal microscope. Approximately 10,000 cells were captured from one agar plate and the mean GFP and mCherry fluorescence signal was quantified for each cell using the ImageJ pipeline as described above. To determine the lowest possible GFP and mCherry signal as a cut-off value, we measured the highest possible green fluorescence from the *prgH::mCherry* single reporter strain and the highest possible red fluorescence from the *csgB::GFP* single reporter strain that were grown on NGM under the same conditions. A background value of 1.0 MFI was subtracted from all images. Cells with mCherry MFI greater than 8.0 were considered as SPI-1 T3SS positive and cells with GFP 97 MFI greater than 6.0 were considered as biofilm positive.

S8: Imaging individual *C. elegans* worms infected with *S.* Typhimurium dual reporter strain D.

Wildtype N2 worms were synchronized and prepared as described above, and for each experiment 60 worms were transferred to a single NGM plate seeded with *S*. Typhimurium 14028 dual reporter strain D. Worms were transferred daily to fresh NGM plates seeded with *Salmonella*. The aim was to image 5 different adult worms at each of 24 h, 48 h, 72 h and 96 h time points. Due to experimental variability, we could not always obtain 5 worms at each time point; therefore, the final imaging data was derived from five individual experiments. Selected worms were removed from the agar, washed with M9 buffer and added to an agarose pad slide with a 5 µL drop of levamisole HCl (5 mM). A coverslip was applied, and the immobilized worms were imaged using a Leica SP8 confocal microscope. Images were taken running down the length of the worm (i.e., front/head region, middle 1, middle 2, back end), and moving up and down in the Z direction while focusing on the intestine. Images were captured using a 63X oil immersion objective lens and

488/561/633 filter, with the Gain set at 1250.0 V, and the frame average set at 6. All the images were manually counted and scored as having red, green or red and green cells.

REFERENCES

Shivak, D.J., MacKenzie, K.D., Watson, N.L., Pasternak, J.A., Jones, B.D., Wang, Y., DeVinney, R., Wilson, H.L., Surette, M.G., and White, A.P. (2016) A modular, Tn7- Based system for making bioluminescent or fluorescent Salmonella and Escherichia coli strains. Appl. Environ. Microbiol. 82, 4931-4943. 10.1128/AEM.01346-16

MacKenzie, K.D., Wang, Y., Shivak, D.J., Wong, C.S., Hoffman, L.J., Lam, S., Kröger, C., Cameron, A.D., Townsend, H.G., Köster, W., and White, A.P. (2015). Bistable expression of CsgD in Salmonella enterica serovar Typhimurium connects virulence to persistence. Infect. Immun. 83, 2312-2326. 10.1128/IAI.00137-15

Palominos, M.F., and Calixto, A. (2020). Quantification of Bacteria Residing in Caenorhabditis elegans Intestine. Bio Protoc. 10(9), e3605. 10.21769/BioProtoc.3605
