# Supplementary material for: Exploring bet-hedging in Salmonella enterica serovar Typhimurium with a dual reporter strain
Source: mSystems. 2026 May 18;11(6):e00177-26. doi: 10.1128/msystems.00177-26 (PMC13289073; doi:10.1128/msystems.00177-26)
Supplement: Reviewer comments [file reviewer-comments.pdf]

AMERICAN SOCIETY FOR MICROBIOLOGY | mSystems®

Exploring Bet-hedging in *Salmonella* enterica serovar Typhimurium with a Dual reporter strain

Michelle Gerber, Murugesan Sivaranjani, Kyle Vincent, Samantha Jelfimow, Dakoda Herman, Melissa Palmer, Chinenye Nnajide, Haley Sanderson, Jessica Sparrow, Brandon Waddell, Neeraj Dhar, Cheng-Wei Wu, and Aaron White

Corresponding Author(s): Aaron White, University of Saskatchewan

Review Timeline:

Submission Date:	February 12, 2026
Editorial Decision:	March 24, 2026
Revision Received:	April 6, 2026
Editorial Decision:	April 13, 2026
Revision Received:	April 14, 2026
Accepted:	April 21, 2026

Editor: Julia Willett

Reviewer(s): The reviewers have opted to remain anonymous.

Transaction Report:

(Note: With the exception of the correction of typographical or spelling errors that could be a source of ambiguity, letters and reports are not edited. The original formatting of letters and referee reports may not be reflected in this compilation.)

DOI: https://doi.org/10.1128/msystems.00177-26

Re: mSystems00177-26 (**Exploring Bet-hedging in *Salmonella* enterica serovar Typhimurium with a Dual reporter strain**)

Dear Dr. Aaron P White:

Thank you for the privilege of reviewing your work. Below you will find my comments, instructions from the mSystems editorial office, and the reviewer comments.

Both reviewers highlight the quality of the work and convincing results. Please respond to their comments, including the point about data not shown.

Please return the manuscript within 60 days; if you cannot complete the modification within this time period, please contact me. If you do not wish to modify the manuscript and prefer to submit it to another journal, notify me immediately so that the manuscript may be formally withdrawn from consideration by mSystems.

Revision Guidelines
To submit your modified manuscript, log into the submission site at https://msystems.msubmit.net/cgi-bin/main.plex. Go to Author Tasks and click the appropriate manuscript title to begin. The information you entered when you first submitted the paper will be displayed; update this as necessary. Note the following requirements:

• Upload point-by-point responses to the issues raised by the reviewers in a file named "Response to Reviewers," NOT in your cover letter.
• Upload a compare copy of the manuscript (without figures) as a "Marked-Up Manuscript" file.
• Upload a clean .DOC/.DOCX version of the revised manuscript and remove the previous version.
• Each figure must be uploaded as a separate, editable, high-resolution file (TIFF or EPS preferred), and any multipanel figures must be assembled into one file.
• Any supplemental material intended for posting by ASM should be uploaded with their legends separate from the main manuscript. You can combine all supplemental material into one file (preferred) or split it into a maximum of 10 files with all associated legends included.

For complete guidelines on revision requirements, see our Submission and Review Process webpage. Submission of a paper that does not conform to guidelines may delay acceptance of your manuscript.

Data availability: ASM policy requires that data be available to the public upon online posting of the article, so please verify all links to sequence records, if present, and make sure that each number retrieves the full record of the data. If a new accession number is not linked or a link is broken, provide mSystems production staff with the correct URL for the record. If the accession numbers for new data are not publicly accessible before the expected online posting of the article, publication may be delayed; please contact production staff (mSystems@asmusa.org) immediately with the expected release date.

Publication Fees: For information on publication fees and which article types are subject to charges, visit our website. If your manuscript is accepted for publication and any fees apply, you will be contacted separately about payment during the production process; please follow the instructions in that e-mail. Arrangements for payment must be made before your article is published.

ASM Membership: Corresponding authors may join or renew ASM membership to obtain discounts on publication fees. Need to upgrade your membership level? Please contact Customer Service at Service@asmusa.org.

The ASM Journals program strives for constant improvement in our submission and publication process. Please tell us how we can improve your experience by taking this quick Author Survey.

Thank you for submitting your paper to mSystems.

Sincerely,
Julia Willett
Editor
mSystems

Reviewer #1 (Comments for the Author):

In this manuscript, Gerber and colleagues explored how the decision to activate biofilm versus virulence transcriptional programs in S. Typhimurium is distributed at a single cell level. To accomplish this, the authors developed a dual reporter system that indicates whether CsgD (biofilm transcriptional activator) and HilA (SPI-1 virulence activator) regulated promoters

are induced within single cells, which was experimentally validated as a reliable tool for their purposes. The authors then tested their dual reporter system in S. Typhimurium biofilm associated and planktonic cells collected from in vitro growth assays. They found that the CsgD activated promoter was singly induced in most cells from biofilms, whereas the HilA activated promoter was singly induced in the largest proportion of planktonic cells. Similar results were observed in three out of four other non-Typhoidal Salmonella strains. Finally, the authors used their dual reporter system to test how biofilm and SPI-1 transcriptional programs are induced during infection using the C. elegans model. During early infection, induction of only the HilA regulated promoter fusion was apparent in most worms. However, as infection progressed, induction of both promoters became apparent in the majority of worms. Altogether, these data support the author's main conclusion that heterogenous induction of biofilm vs virulence programs occurs during gut infection, while also demonstrating the utility of the genetic tool they developed to address their overarching question.

Altogether, this is a very well executed and rigorous study elegantly demonstrating the concept of bet-hedging among Salmonella populations during gastrointestinal infection. All interpretations and conclusions are supported by the data and images presented, and acknowledgements are made regarding important limitations such as the roles of temperature and osmolarity in impacting the induction of the biofilm and SPI-1 programs at the single cell level.

Moderate point:

1)In the discussion, it may be worth noting whether other regulatory networks exist in Salmonella that also contribute to biofilm formation in addition to CsgD and curli fimbriae. Are there regulators of other matrix components such as EPS or other fimbriae that contribute to Salmonella and that could be worthy of future study in terms of bet hedging?

Reviewer #2 (Comments for the Author):

In this manuscript the authors construct a dual transcriptional reporter to a major biofilm and virulence component of Salmonella typhimurium ATCC 14028 in order to investigate the biofilm/acute virulence switch eventually in a C. elegans infection model. The work is performed well and the results are convincing, however, to place the results in a more general context is missing. The dual role of amyloid curli in biofilm formation and virulence is not addressed. Also, regulation of the second component of the extracellular matrix regulated by csgD, the exopolysaccharide cellulose, cannot be readily addressed by transcriptional fusions although involved in virulence modulation. Therefore, as the correlations are not absolute, ttese aspects need be mentioned in the discussion.

Specific comments
l. 109: Curli can also trigger cell invasion
l.154: Why is construct A not shown? Is there any rational why these reporters behave differently?
l.173: grown under which conditions? The degree of biofilm formation (and virulence) is highly dependent on the growth conditions.
l.180: While these numbers are exact, the ratio of biofilm versus planktonic cells and the ration of the different cell types might be highly dependent on the experimental conditions. Bacteria harbouring the dual reporter fusions can be grown under different conditions and in different mutant backgrounds to strengthen the reliability of the results obtained from the reporter transcriptional fusions.
l.196: Is the dual reporter strain of S. typhimurium ATCC 14028 equally virulent as the wild type non-motified strain?
l.244: Please show these data.
l.256: It would be highly preferable here to correlate the biofilm formation capacity of the different strains with the results from the dual reporter fusions. For example, strain LT2 is known to be impaired in the stress sigma factor RpoS and considered to be non-virulent which should affect biofilm formation and virulence etc.
l.273: In case of biofilm formation, save energy perhaps.
l.334: normal? Standard conditions

RESPONSE TO REVIEWERS (mSystems00177-26) **(Our Responses in Blue)**

Reviewer #1:

Moderate point:
1) In the discussion, it may be worth noting whether other regulatory networks exist in Salmonella that also contribute to biofilm formation in addition to CsgD and curli fimbriae. Are there regulators of other matrix components such as EPS or other fimbriae that contribute to Salmonella and that could be worthy of future study in terms of bet hedging?

This is a difficult question to answer. In Salmonella serovar Typhi, there is noted biofilm formation on gallstones in humans, and this is known to be independent of CsgD. Therefore, other components are involved in biofilm formation, but we are unsure if biofilms are bistably expressed or not. We have added one reference – Gonzalez et al. Front Cell Infect Microbiol 2024 – and added a statement in the Discussion.

Reviewer #2:

The work is performed well and the results are convincing, however, to place the results in a more general context is missing. The dual role of amyloid curli in biofilm formation and virulence is not addressed. Also, regulation of the second component of the extracellular matrix regulated by csgD, the exopolysaccharide cellulose, cannot be readily addressed by transcriptional fusions although involved in virulence modulation. Therefore, as the correlations are not absolute, ttese aspects need be mentioned in the discussion.

Specific comments
l. 109: Curli can also trigger cell invasion
There isn't a lot of evidence or information that curli are invasion factors. They bind to TLR2 and stimulate autoimmunity and are expressed during infections, but to my knowledge there isn't a lot of information about them contributing to invasion. Is there a specific paper or papers that the reviewer is referring to?

l.154: Why is construct A not shown? Is there any rational why these reporters behave differently?
The dual reporter strain built from Construct A did not show the proper biofilm phenotype (Fig. S3), indicating that the CsgD pathway was impaired, and did not grow well on Kanamycin. We don't know the exact reason for this. Our best guess is that the prgH-mCherry reporter is facing towards the chromosomal glmS gene in constructs A and C and this causes issues, but we have never proven this. We designed all four constructs (A,B,C,D) for precisely this reason, anticipating that each dual reporter strain may not behave the same. We were not even sure if we could build all constructs due to DNA regions being incompatible

l.173: grown under which conditions? The degree of biofilm formation (and virulence) is highly dependent on the growth conditions.

For this study, we are following the growth conditions that we outlined in MacKenzie et al. 2015, which was used as the original way to show population splitting in the S. Typhimurium 14028 cultures. We agree with the reviewer that different conditions could change the proportions of biofilm cells vs. single cells, but that was not the point of this study. At first, we thought we should stick to the original conditions where RNA-seq was performed, to make our comparisons most relevant to the information we had gathered previously. Now that this dual reporter strain has been built and characterized we can investigate different growth conditions and how that changes the bet hedging process.

l.180: While these numbers are exact, the ratio of biofilm versus planktonic cells and the ration of the different cell types might be highly dependent on the experimental conditions. Bacteria harbouring the dual reporter fusions can be grown under different conditions and in different mutant backgrounds to strengthen the reliability of the results obtained from the reporter transcriptional fusions.

We agree with the reviewer's comments here - due to the amount of work that was performed to quantify the different cell types, we did not pursue growth under other conditions to see if the population proportions will change. This could be an area of future research.

l.196: Is the dual reporter strain of S. typhimurium ATCC 14028 equally virulent as the wild type non-motified strain?

We have not explicitly tested if the dual reporter strain "D" is equally virulent to the un-marked wildtype 14028 strain. We did compare the virulence in worms of our dual reporter D to wildtype 14028 that was tagged with a constitutive luciferase reporter, inserted at the the same attTn7 site in the chromosome; these strains had similar virulence. We have used luciferase-tagged strains in the past to perform competitive index infections in mice (MacKenzie et al., 2015; Shivak et al., 2016; Sokaribo et al., 2021) and they are still virulent. However, we are never competing pure wildtype against a tagged strain.

We anticipate that the dual reporter tagged strain would be less virulent than an untagged strain. Based on what a collaborator has shared with us, insertion into the attTn7 site does alter virulence slightly compared to a wildtype strain, but this has never been published

l.244: Please show these data.

We have added supplementary Figure S6 to show two examples of infected worms that we think are likely to contain non-fluorescent Salmonella cells – one worm at 72h and one at 96h.

l.256: It would be highly preferable here to correlate the biofilm formation capacity of the different strains with the results from the dual reporter fusions. For example, strain LT2 is known to be impaired in the stress sigma factor RpoS and considered to be non-virulent which should affect biofilm formation and virulence etc.

We have previously quantified biofilm formation for SL1344 and D23580 (MacKenzie et al., PLoS Genetics 2019), but not for LT2. I agree with the reviewer that LT2 is likely impaired. We performed the experiments in Figure 8 to show that this bet-hedging system works in other non-typhoidal Salmonella strains, it was not to compare the proportions of the different cell types per se. We predict that the ratio is something inherent to the strains, likely influenced by their evolutionary history, however, before we make a big statement like this, we would need to do more testing.

l.273: In case of biofilm formation, save energy perhaps.

We have added “and save energy” to Line 273

l.334: normal? Standard conditions

we replaced “normal” with “standard” as suggested

Re: mSystems00177-26R1 (**Exploring Bet-hedging in *Salmonella* enterica serovar Typhimurium with a Dual reporter strain**)

Dear Dr. Aaron P White:

Thank you for the privilege of reviewing your work. Below you will find my comments, instructions from the mSystems editorial office, and the reviewer comments.

Please clarify whether the instance of "data not shown" on line 227 of the marked up manuscript is the same as what is now represented in Figure S6. If not, please address this other instance of data not shown. Please also comment on the suggestion from reviewer 2 (in the first paragraph of the review of the initial manuscript) about cellulose.

Please return the manuscript within 60 days; if you cannot complete the modification within this time period, please contact me. If you do not wish to modify the manuscript and prefer to submit it to another journal, notify me immediately so that the manuscript may be formally withdrawn from consideration by mSystems.

Revision Guidelines
To submit your modified manuscript, log into the submission site at https://msystems.msubmit.net/cgi-bin/main.plex. Go to Author Tasks and click the appropriate manuscript title to begin. The information you entered when you first submitted the paper will be displayed; update this as necessary. Note the following requirements:

• Upload point-by-point responses to the issues raised by the reviewers in a file named "Response to Reviewers," NOT in your cover letter.
• Upload a compare copy of the manuscript (without figures) as a "Marked-Up Manuscript" file.
• Upload a clean .DOC/.DOCX version of the revised manuscript and remove the previous version.
• Each figure must be uploaded as a separate, editable, high-resolution file (TIFF or EPS preferred), and any multipanel figures must be assembled into one file.
• Any supplemental material intended for posting by ASM should be uploaded with their legends separate from the main manuscript. You can combine all supplemental material into one file (preferred) or split it into a maximum of 10 files with all associated legends included.

For complete guidelines on revision requirements, see our Submission and Review Process webpage. Submission of a paper that does not conform to guidelines may delay acceptance of your manuscript.

Data availability: ASM policy requires that data be available to the public upon online posting of the article, so please verify all links to sequence records, if present, and make sure that each number retrieves the full record of the data. If a new accession number is not linked or a link is broken, provide mSystems production staff with the correct URL for the record. If the accession numbers for new data are not publicly accessible before the expected online posting of the article, publication may be delayed; please contact production staff (mSystems@asmusa.org) immediately with the expected release date.

Publication Fees: For information on publication fees and which article types are subject to charges, visit our website. If your manuscript is accepted for publication and any fees apply, you will be contacted separately about payment during the production process; please follow the instructions in that e-mail. Arrangements for payment must be made before your article is published.

ASM Membership: Corresponding authors may join or renew ASM membership to obtain discounts on publication fees. Need to upgrade your membership level? Please contact Customer Service at Service@asmusa.org.

The ASM Journals program strives for constant improvement in our submission and publication process. Please tell us how we can improve your experience by taking this quick Author Survey.

Thank you for submitting your paper to mSystems.

Sincerely,
Julia Willett
Editor
mSystems

RESPONSE TO REVIEWERS (mSystems00177-26 – revision 2) **(Our Responses in Blue)**

Reviewer #2:

Also, regulation of the second component of the extracellular matrix regulated by csgD, the exopolysaccharide cellulose, cannot be readily addressed by transcriptional fusions although involved in virulence modulation.

Specific comments
L. 324 – 327
We added a few statements mentioning cellulose and how the role is complex and unknown inside C. elegans.

l.226: Please show these data.
We added another worm picture at 24 hours post-Salmonella infection to Figure S6. We missed that there were two references of (Data not shown) within the same paragraph. Now Figure S6 shows images from 24, 72 and 96h post infection and in each case we think that non-fluorescent *S.* Typhimurium cells are present.

Re: mSystems00177-26R2 (**Exploring Bet-hedging in *Salmonella* enterica serovar Typhimurium with a Dual reporter strain**)

Dear Dr. Aaron P White:

Your manuscript has been accepted, and I am forwarding it to the ASM production staff for publication. Your paper will first be checked to make sure all elements meet the technical requirements. ASM staff will contact you if anything needs to be revised before copyediting and production can begin. Otherwise, you will be notified when your proofs are ready to be viewed.

Data Availability: ASM policy requires that data be available to the public upon online posting of the article, so please verify all links to sequence records, if present, and make sure that each number retrieves the full record of the data. If a new accession number is not linked or a link is broken, provide production staff with the correct URL for the record. If the accession numbers for new data are not publicly accessible before the expected online posting of the article, publication may be delayed; please contact ASM production staff immediately with the expected release date.

Publication Fees: For information on publication fees and which article types have charges, please visit our website. We have partnered with Copyright Clearance Center (CCC) to collect author charges. If fees apply to your paper, you will receive a message from no-reply@copyright.com with further instructions. For questions related to paying charges through RightsLink, please contact CCC at ASM_Support@copyright.com or toll free at +1-877-622-5543. CCC makes every attempt to respond to all emails within 24 hours.

ASM Membership: Corresponding authors may join or renew ASM membership to obtain discounts on publication fees. Need to upgrade your membership level? Please contact Customer Service at Service@asmusa.org.

Embargo Policy: A press release may be issued as soon as the manuscript is posted on the mSystems Latest Articles webpage. The corresponding author will receive an email with the subject line "ASM Journals Author Services Notification" when the article is available online.

Cover Image Submissions: If you would like to submit a potential Cover Image, please email a file and a short legend to msystems@asmusa.org. Please note that we can only consider images that (i) the authors created or own and (ii) have not been previously published. By submitting, you agree that the image can be used under the same terms as the published article. Image File requirements: TIF/EPS, 7.5 inches wide by 8.25 inches tall (at least 2,250 pixels wide by 2,475 pixels tall), minimum 300 dpi resolution (600 dpi preferred), RGB, and no figure elements, e.g., arrows or panel labels. The legend should be a short description of the image, 1-2 sentences recommended. Please download and use this interactive template in Adobe to ensure that your proposed cover image meets our size requirements (https://journals.asm.org/pb-assets/pdf-text-excel-files/ASM-Interactive-Sizing-Cover-Template-1715689791.pdf).

Thank you for submitting your paper to mSystems.

Sincerely,
Julia Willett
Editor
mSystems
